# AS3MT-mediated tolerance to arsenic evolved by multiple independent horizontal gene transfers from bacteria to eukaryotes

**DOI:** 10.1371/journal.pone.0175422

**Published:** 2017-04-20

**Authors:** Michael Palmgren, Karin Engström, Björn M. Hallström, Karin Wahlberg, Dan Ariel Søndergaard, Torbjörn Säll, Marie Vahter, Karin Broberg

**Affiliations:** 1 Unit of Metals & Health, Institute of Environmental Medicine, Karolinska Institutet, Stockholm, Sweden; 2 Centre for Membrane Pumps in Cells and Disease—PUMPKIN, Department of Plant and Environmental Sciences, University of Copenhagen, Frederiksberg C, Denmark; 3 Laboratory Medicine, Division of Occupational and Environmental Medicine, Lund University, Lund, Sweden; 4 Science for Life Laboratory, KTH—Royal Institute of Technology, Stockholm, Sweden; 5 Center for Bioinformatics (BiRC), Aarhus University, Aarhus C, Denmark; 6 Department of Biology, Lund University, Lund, Sweden; Columbia University, UNITED STATES

## Abstract

Organisms have evolved the ability to tolerate toxic substances in their environments, often by producing metabolic enzymes that efficiently detoxify the toxicant. Inorganic arsenic is one of the most toxic and carcinogenic substances in the environment, but many organisms, including humans, metabolise inorganic arsenic to less toxic metabolites. This multistep process produces mono-, di-, and trimethylated arsenic metabolites, which the organism excretes. In humans, arsenite methyltransferase (AS3MT) appears to be the main metabolic enzyme that methylates arsenic. In this study, we examined the evolutionary origin of AS3MT and assessed the ability of different genotypes to produce methylated arsenic metabolites. Phylogenetic analysis suggests that multiple, independent horizontal gene transfers between different bacteria, and from bacteria to eukaryotes, increased tolerance to environmental arsenic during evolution. These findings are supported by the observation that genetic variation in *AS3MT* correlates with the capacity to methylate arsenic. Adaptation to arsenic thus serves as a model for how organisms evolve to survive under toxic conditions.

## Introduction

Organisms adapt to toxic environments. Bacteria and plants have been shown to undergo genetic adaptations that allow them to withstand toxicants in water and soil [1–3]. In eukaryotes, already existing resistance genes can be potentiated after duplication events that increase gene copy number [2] or when mutations introduce alleles that enable the organism to better tolerate the toxicant [4], but little is known about how tolerance to toxic environments actually evolves. Based on the findings that a close homolog of the bacterial arsenic transport gene *ArsB* and a homolog of the mercuric reductase gene are present in the genome of the extremophile red alga *Galdieria sulphuraria*, it was suggested that horizontal gene transfers (HGTs) from prokaryotes to eukaryotes provided a further mechanism of adaptation to toxic environments [5]. In that study, no other eukaryotic homologs of *ArsB* or mercuric reductase showed signs of HGT and, therefore, the extent to which HGT provides a mechanism for the development of tolerance to toxic environments in eukaryotes remains an open question.

**Fig 1:**
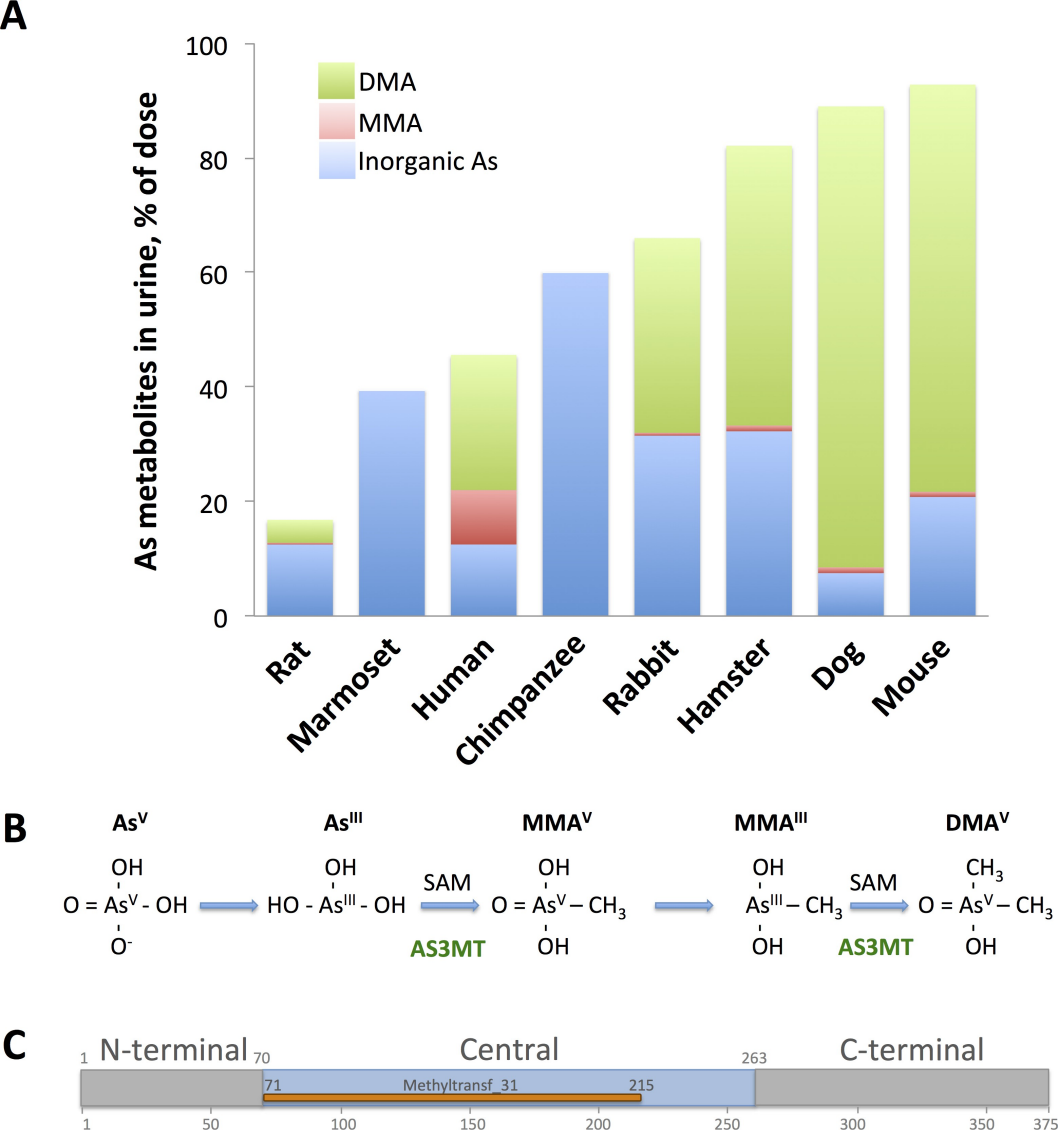
**(A). The arsenic methylation capacity differs in different animal species.** Urinary excretion of arsenic metabolites (InorgAs, inorganic arsenic; MMA, methylarsonic acid; and DMA, dimethylarsinic acid) in different species 2–4 days after ingestion of a single dose of arsenate. Derived from Vahter [13]. **(B) Proposed pathway of biomethylation of arsenic in mammalian systems.** Human arsenic (+3 oxidation state) methyltransferase (AS3MT) catalyses methylation of trivalent arsenic (As(III)) to monomethylarsonate (MMA(V)) and dimethylarsinic acid (DMA(V)) with S-adenosylmethionine (SAM) as the methyl donor. (C) Schematic overview of primary structure of human AS3MT. The enzyme of 365 amino acid residues is organized in three domains [34]: A N-terminal domain (residues 1–70), a central domain (residues 71–263) with the methyltransferase domain (Methyltransf_31; residues 71–215), and a C-terminal domain (residues 264–315).

Inorganic arsenic, one of the most potent toxicants in nature, occurs in elevated concentrations in groundwater and soil in many parts of the world. In humans, numerous epidemiological studies have shown associations between arsenic exposure through ingestion or inhalation and increased risk of cancer, severe non-malignant diseases, such as diabetes, lung and cardiovascular diseases [6–9]; and child morbidity and mortality [10,11]. Humans show large variation in their susceptibility to arsenic, possibly due to differences in their ability to metabolise this toxic element [12]. Most mammals metabolise inorganic arsenic via methylation to methylarsonic acid (MMA) and dimethylarsinic acid (DMA), although there are major differences in arsenic methylation capacity between species [12,13], as shown for animals in Fig 1A. Arsenic is likely methylated via alternating the reduction of pentavalent arsenate to trivalent arsenite with the addition of methyl groups, although the exact sequence of events remains a matter of debate (Fig 1B) [14–16]. The main enzyme catalysing the methylation is arsenite methyltransferase (Fig 1C) [called AS3MT in animals and ArsM in microorganisms, the latter of which is part of the arsenic resistance *ars* operon [17–23] and the only reported function of AS3MT is to methylate inorganic arsenite. Some bacteria and fungi can methylate DMA further to volatile trimethylarsine (TMA) [14,24].

Arsenic methylation is advantageous to organisms; for example, mice with a knock-out mutation of *AS3MT* retain much higher concentrations of arsenic in different tissues than do wild-type mice [25]. In humans, methylation of arsenic reduces the retention of arsenic in the body [12,26], supporting the idea that methylation of arsenic to DMA in mammals (and further to TMA in bacteria) acts as a detoxification mechanism. However, the first methylated product, MMA, especially in its trivalent form, is considered to be more toxic and carcinogenic than arsenite [27–30]. Interestingly, in humans MMA mostly is excreted (Fig 1) and a higher fraction of MMA in urine has been associated with a higher risk of different cancers, skin lesions, and cardiovascular effects [31]. Thus, it is surprising that AS3MT-catalyzed methylation of inorganic arsenic yields products that are more reactive and toxic than the parent compound [29,32]. Although AS3MT catalyses the formation of toxic metabolites, it is still present and conserved in multiple different kingdoms. Thomas et al. [33] suggested that the capacity to methylate arsenic has provided an adaptive advantage throughout evolution. The persistence of AS3MT in species would thus depend on the balance of the advantageous and deleterious effects of methylation of arsenic compounds.

Can tracing the evolution of AS3MT explain how tolerance to arsenic evolved in nature? In this study, we addressed this question by combining an analysis of the evolutionary origin of AS3MT with an analysis of the relationship between *AS3MT* genotype and phenotype. We show that AS3MT is essential for arsenic methylation capacity and present evidence that HGTs from prokaryotes to eukaryotes underlie adaptations to arsenic, thereby providing insight into how toxic environments shape the evolution of eukaryotes.

## Materials and methods

### Phylogenetic analysis of AS3MT

Sequences with significant similarity (Expect value of <e^−50^) to the well-characterized *H*. *sapiens* AS3MT were identified in the NCBI protein database using the Basic Local Alignment Search Tool (BLAST) program (http://blast.ncbi.nlm.nih.gov/). In the case of non-animal eukaryotes, all sequences with significant similarity (Expect value of <e^−50^) to AS3MT were selected. Among bacterial sequences, a subset was selected that showed the highest level of sequence similarity to AS3MT. Among animals, searches were restricted to organisms representing major phyla. The AS3MT homolog in the charophyte *Klebsormidium flaccidium* was identified through a BLAST search at the plantmorphogenesis server (http://www.plantmorphogenesis.bio.titech.ac.jp/~algae_genome_project/klebsormidium/klebsormidium_blast.html). For all eukaryotic kingdoms and archaea, additional BLAST searches were carried out in the NCBI protein database using the sequence in that kingdom with highest similarity to human AS3MT as query. All AS3MT homologs selected were assumed to be orthologs. As most genomes in the NCBI protein database are still in draft form, the predicted AS3MTs often did not represent complete proteins; these partial sequences were removed following alignment. Furthermore, since S-adenosylmethionine (SAM) is the main methyl donor in arsenic methylation [34], sequences without signature motifs characteristic of SAM-dependent methyltransferases [35] were not considered to be arsenic methyl transferases and consequently eliminated from the data set. In subsequent searches a lower cut-off (<e^1^) was employed in order to identify AS3MT variants in the selected species that were missed with the higher threshold. This analysis did not result in the identification of new sequences likely to be arsenic methyltransferases.

**Fig 2:**
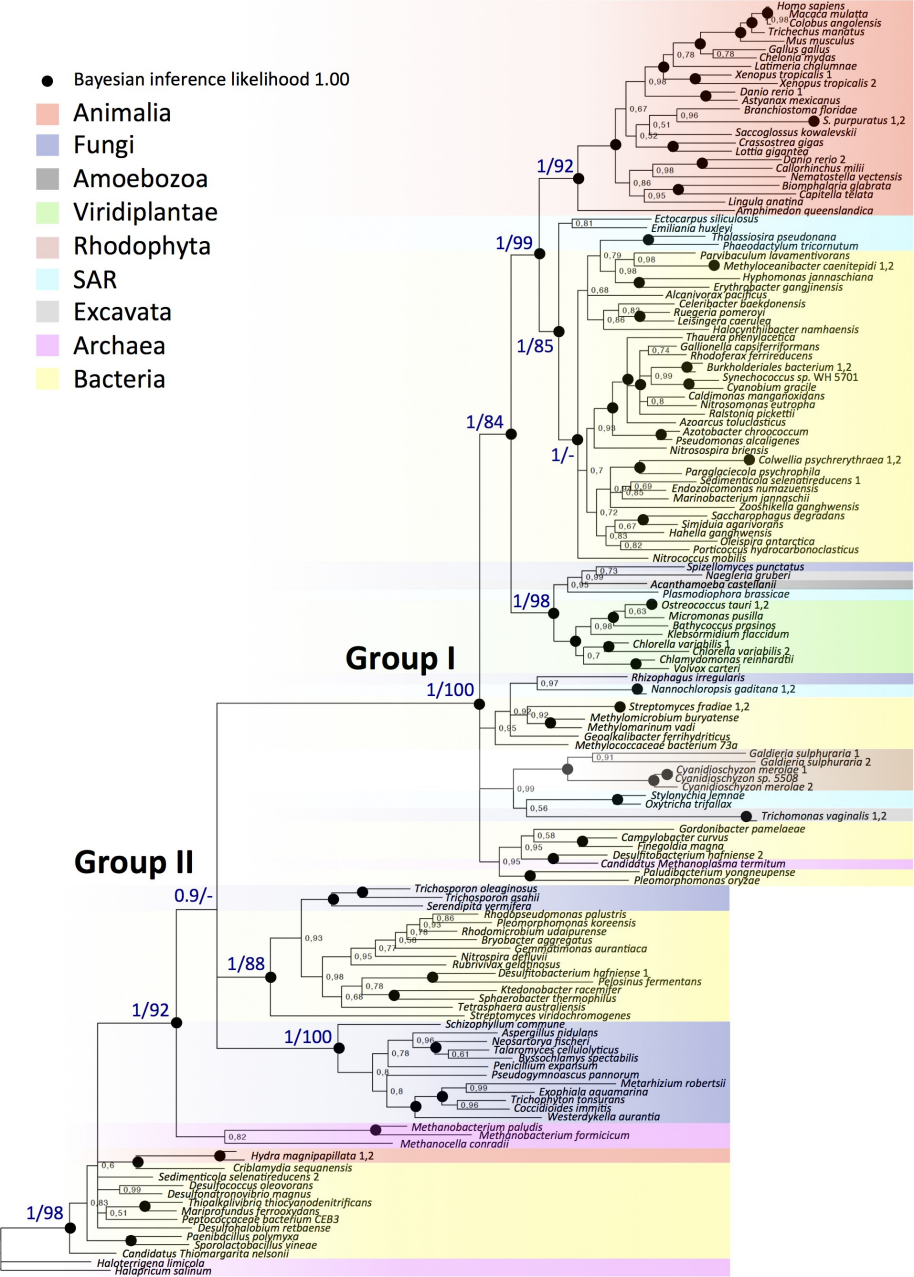
Phylogenetic analysis of AS3MT proteins from animal, fungal, green and red algal, and other eukaryotic lineages, as well as archaeal and bacterial lineages demonstrates that AS3MT is present in a range of kingdoms. The tree shown is the consensus tree derived by Bayesian inference using MrBayes, as described in Experimental procedures. MrBayes posterior probabilities are shown for branches with >0.5 MrBayes posterior probability support; branches with a value of 1 are shown by a filled circle. Bootstrap values of the same tree, derived by maximum likelihood using the program RAxML, are shown at key branches. AS3MT is phylogenetically split into one major group (I) divided into one subgroup of bacteria, SAR (stramenopiles, alveolata, rhizaria), and animals; and another major group (II) of bacteria, archaea, ascomycote and basidiomycote fungi, and *Hydra magnipapillata*. The species names and the database accession numbers are explained in S1 Table. Species that have two isoforms are shown with numbers 1 and 2 for the respective isoform.

Protein alignment was performed with MUSCLE [36]. All positions containing gaps or ambiguous data were eliminated, leaving a total of 189 positions in the final data set. In the analysis of the individual AS3MT domains, all positions containing gaps or ambiguous data were retained in the data sets. The evolutionary history was inferred assuming an LG [37] + INVGAMMA model, as identified by ProtTest [38]. Phylogenetic analyses were subsequently conducted using Bayesian inference and maximum likelihood methods. Bayesian inference was performed with MrBayes 3.2.6 [39] and maximum likelihood analyses with RAxML 8.2.4 [40] and MEGA6 [41]. MrBayes analysis was performed using the following settings: eight chains of Markov chain Monte Carlo iterations and heated parameter of 0.05 with trees sampled every 100 generations. The unrooted consensus tree of the MrBayes analysis was selected for presentation. The average standard deviations of split frequencies at termination of the analysis were, after 2,960,000 generations, 0.009967 for the AS3MT tree in Fig 2. AS3MT trees of group I and II (S4 Fig) were both run for 4,000,000 generations and resulted in average standard deviations of split frequencies at termination of 0.008648 for group I and of 0.004565 for group II. In the RAxML analysis, clade robustness was assessed with 100 rapid bootstrap inferences followed by thorough analysis of maximum likelihood. In the MEGA6 analysis, bootstrap values were inferred from 1000 replicates to obtain statistical support for the placement of nodes [42]. Accession numbers are listed in S1 Table.

### Comparison of unconstrained versus constrained AS3MT trees

For comparisons of constrained (when fungal and animal protein sequences were forced to cluster monophyletically) versus unconstrained trees RAxML was used. All RAxML analyses were performed using the same model as used for MrBayes (PROTGAMMILGF). First, the branch lengths of the MrBayes tree were recalculated in RAxML using the same model as for MrBayes, using the “optimize model parameters+branch lengths for given input tree” (-f e) mode in RAxML. An unconstrained RAxML tree was also created. Then, two different constrained trees were determined by providing RAxML with multifurcating constraint trees (using the–g option) where all fungal and animal species were forced into monophyly. Finally, the MrBayes tree (with recalculated branch lengths) was compared to 1) the unconstrained RAxML tree and 2) the constrained RAxML tree, in a log likelihood test (Shimodaira-Hasegawa test), using the–f H option in RAxML. The tree with a lower likelihood was scored as the worst and thus less likely to be true.

### Comparison of different proteins

Sequences of SERCA2-like calcium pumps and ATP7A-like copper pumps were extracted by BLAST searches from the genomes of the same 134 species used to analyse the AS3MT-like sequences. For each species, BLAST searches were carried out with human SERCA2 (ATP2A2; P16615) and ATP7A (Q04656). The highest hit in each species was selected and inspected manually for conserved sequence motifs characteristic of P-type SERCA [43] and copper [44] pumps. The resulting data set contained SERCA2-like calcium pumps and ATP7A-like copper pumps from 92 and 113 species, respectively. Initial tree(s) for AS3MT, SERCA2, and ATP7A for the heuristic search were obtained automatically by applying the Neighbor-Joining and BioNJ algorithms to a matrix of pairwise distances estimated using the JTT model (for simplicity when analysing three different proteins), and then selecting the topology with the superior log likelihood value. All positions containing gaps and ambiguous data were eliminated. To obtain statistical support for the placement of nodes, bootstrap values were inferred from 1000 replicates [42]. Evolutionary analyses were conducted in MEGA6 [41]. Accession numbers are listed in S1 Table.

### Intron presence and GC content analysis

Reference genomes, annotations (GFF3), and coding sequences (CDS) were downloaded for each species from Ensembl Genomes (www.ensembl.org). The number of introns was counted from the GFF3 file, and in cases where no introns were annotated a BLASTX search was performed against the genome sequence to confirm the lack of introns. GC content for all coding sequences was determined for each relevant species and the mean and standard deviation were calculated and compared to the GC content of *AS3MT*.

### Correlation of genotype with arsenic metabolism capacity phenotype

The alignment between human AS3MT and selected other species was evaluated using protein BLAST (pBLAST) at NCBI [45,46] employing default parameters. First, the alignment for the whole AS3MT protein was evaluated. Second, the alignment for the conserved methyltransferase domains in AS3MT was evaluated. The domains were 1) AdoMet_MTases; abbreviation of S-AdenosylMethionine-dependent MethylTransferases (SAM or AdoMet-MTase), class I (cd02440) and 2) Methyltransf_31; abbreviation of the Methyltransferase domain. The impact of non-synonymous substitutions on protein function and structure were predicted using SIFT (J. Craig Venter Institute: sift.jcvi.org) and Polyphen (Harvard. PolyPhen-2. Internet: genetics.bwh.harvard.edu/pph2). Further, the alignment between the human *AS3MT* gene and the *AS3MT* homologs from other species (S5 Table) was evaluated using BLASTn (optimized for more dissimilar sequences) and discontiguous Mega BLAST (optimized for somewhat similar sequences), as recommended when comparing across species.

## Results

### AS3MT is widespread in nature but its phylogeny is inconsistent with vertical descent

To elucidate the origin of arsenic metabolism, we searched for homologs of the human AS3MT protein from sequenced genomes. The data set was comprised of 150 sequences of homologs of the human AS3MT protein from 134 species. We identified AS3MT homologs in multiple species of bacteria, archaea, red and green algae, fungi, animals, and other eukaryotic lineages, showing that AS3MT is widespread in nature. However, we did not find genes encoding AS3MT in genomes of insects, arachnids, flatworms, roundworms, and crustaceans. Similarly, angiosperms (seed plants) and bryophytes (mosses) lack homologs of AS3MT.

Very few species have more than one *AS3MT* gene (Fig 2), suggesting that duplication of *AS3MT* was rare during evolution. This observation suggests that *AS3MT* encodes an enzyme involved in a single process (e.g., the metabolism of a single compound) that takes place in different cell types and during different life stages, as several isoforms would be expected in each species if this were not the case. Moreover, as several phyla exist in which some species carry AS3MT but others not, the enzyme is not essential to eukaryotic life, but is beneficial enough to be maintained in the genome. Most of the above-mentioned organisms harboring *AS3MT* have not been analysed for their capacity to methylate arsenic.

We subsequently performed a phylogenetic analysis of AS3MT homologs in 134 species (S1 Table) by aligning the sequence of human AS3MT with the homologous sequences identified by BLAST searches of the fully sequenced genomes of evolutionarily representative species. To obtain maximum statistical support for the phylogenetic analysis, we conducted both Bayesian inference and maximum likelihood analyses. We found statistical evidence for two large monophyletic groups of AS3MT homologs (unrooted consensus tree in Fig 2, analysis based on Bayesian inference analysis with MrBayes and maximum likelihood analysis with RAxML and S1 Fig, analysis based on maximum likelihood analysis with MEGA6).

The first group (I in Fig 2) was divided into two subgroups. Whereas one subgroup was composed of AS3MT homologs derived from bacteria (alpha, beta, and gamma proteobacteria and cyanobacteria) that cluster with AS3MT homologs from eukaryotic species of the SAR (Stramenopiles, Alveolata, Rhizaria) supergroup, exemplified by the stramenopile diatom *Phaeodactylum tricornutum* (Fig 2), the other subgroup was composed of AS3MT homologs from animals, ranging from primates to cnidarians and poriferans, the latter two of which are represented by the starlet sea anemone *Nematostella vectensis* and the sponge *Amphimedon queenslandica*, respectively. The second major phylogenetic group, group (II), consisted of three major branches: one that included AS3MT homologs from phylogenetically diverse bacteria (fibrobacteria, beta and alpha proteobacteria, actinobacteria, firmicutes, and chloroflexi) and basidiomycote fungi; one composed of AS3MT homologs from ascomycete fungi; and one composed of homologs from bacteria (firmicutes, gamma, zeta and delta/epsilon proteobacteria), archaea (euryarchaeota), and two isoforms from the cnidarian *Hydra magnipapillata*.

Apart from the AS3MT homologs in animals having different evolutionary origins (compare the clustering of *H*. *magnipapillata* with that of other animal species in Fig 2), AS3MT homologs in fungi also showed an evolutionarily unexpected and patchy distribution in the tree. Whereas AS3MT sequences of ascomycote and basidiomycote fungi were closely related to those of bacteria and clustered in group II, the two other fungal homologs of AS3MT, i.e., *Spizellomyces punctatus*, which belongs to the phylum *Chytridiomycota*, a very early diverging fungal lineage, and *Rhizophagus irregularis*, which belongs to a lineage of fungi that form mycorrhizal symbioses with plants, were found in group I. AS3MT homologs from archaea (methanobacteria and halobacteria) mainly clustered in group II along with bacteria, fungi, and *H*. *magnipapillata*, but one species (*Candidatus methanoplasma termitum*) clustered with bacteria in group I.

Next, we analysed the phylogeny of the three individual AS3MT domains (as defined by Ajees and Rosen [34]; Fig 1C). Using MrBayes analysis, we found that phylogenetic unrooted trees based on the central and N-terminal domains, which constitute the S-adenosylmethionine (SAM) and arsenic-binding regions, respectively, are similar to each other, and moreover, are similar to trees based on the whole AS3MT protein (S2 and S3 Figs). We interpret this finding as evidence that AS3MT did not simply evolve from other methyltransferases by a few point mutations in its catalytic core, but rather that the specific function of AS3MT required the coordinated evolution of a major part of the protein. The C-terminal domain was not conserved (data not shown), suggesting that this domain, which is not subject to evolutionary pressure, lacks specific functions, as discussed by Ajees and Rosen [34].

**Fig 3:**
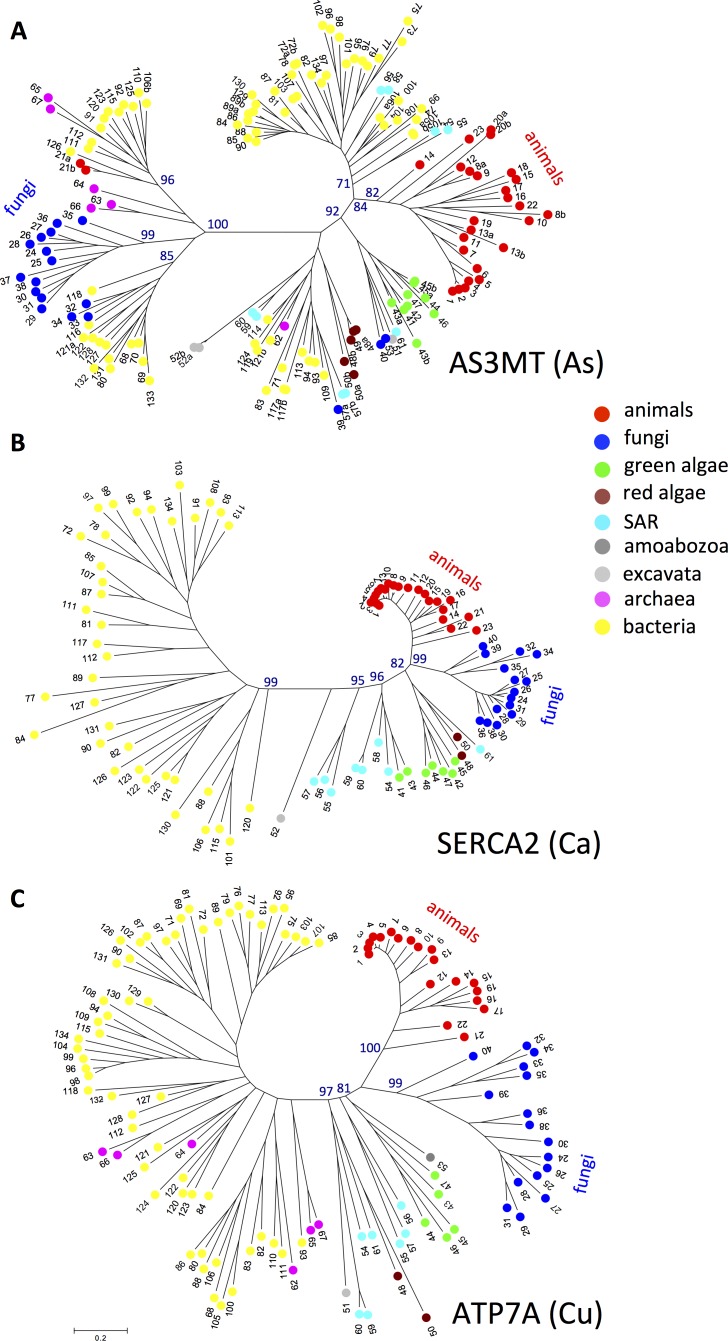
AS3MT shows very different phylogenetic relationships compared with other proteins in the same species. Comparison of the phylogenetic trees based on AS3MT (A), the calcium pump SERCA2 (B), and the copper pump ATP7A (C) in the same species (indicated with numbers). Numbers refer to species as follows: **Animals**: 1, *Homo sapiens*; 2, *Macaca mulatta*; 3, *Colobus angolensis palliatus*; 4, *Trichechus manatus latirostris*; 5, *Mus musculus*; 6, *Gallus gallus*; 7, *Chelonia mydas*; 8, *Danio rerio*; 9, *Astyanax mexicanus*; 10, *Callorhinchus milii*; 11, *Latimeria chalumnae*; 12, *Branchiostoma floridae*; 13, *Xenopus tropicalis*; 14, *Saccoglossus kowalevskii*; 15, *Capitella telata*; 16, *Crassostrea gigas*; 17, *Lottia gigantea*; 18, *Biomphalaria glabrata*; 19, *Lingula anatina*; 20, *Strongylocentrotus purpuratus*; 21, *Hydra magnipapillata*; 22, *Nematostella vectensis*; 23, *Amphimedon queenslandica;* **Fungi**: 24, *Aspergillus nidulans FGSC A4*; 25, *Penicillium expansum*; 26, *Neosartorya fischeri NRRL 181*; 27, *Talaromyces cellulolyticus*; 28, *Byssochlamys spectabilis No*. *5*; 29, *Trichophyton tonsurans CBS 112818*; 30, *Exophiala aquamarina CBS 119918*; 31, *Coccidioides immitis RS*; 36, *Pseudogymnoascus pannorum VKM F-4514*; 37, *Westerdykella aurantiaca*; 38, *Metarhizium robertsii ARSEF 23*; 32, *Trichosporon oleaginosus*; 33, *Serendipita vermifera MAFF 305830*; 34, *Trichosporon asahii var*. *asahii CBS 8904*; 35, *Schizophyllum commune H4-8*; 36, *Pseudogymnoascus pannorum VKM F-4514*; 37, *Westerdykella aurantiaca*; 38, *Metarhizium robertsii ARSEF 23*; 39, *Rhizophagus irregularis*; 40, *Spizellomyces punctatus DAOM BR117*; **Green algae**: 41, *Chlamydomonas reinhardtii*; 42, *Volvox carteri f*. *nagariensis***;** 43, *Chlorella* variabilis; 44, *Micromonas pusilla CCMP1545***;** 45, *Ostreococcus tauri***;** 46, *Bathycoccus prasinos*; **Charophyta**: 47, *Klebsormirdium flaccidum***; Red algae**: 48, *Cyanidioschyzon merolae* strain 10D; 49, *Cyanidioschyzon sp*. *5508*; 50, *Galdieria sulphuraria***; Excavata**: 51, *Naegleria gruberi NEG-M***;** *52*, *Trichomonas vaginalis G3***; Amoebozoa;** 53, *Acanthamoeba castellanii str*. *Neff***; SAR:** 54, *Thalassiosira pseudonana CCMP1335***;** 55, *Phaeodactylum tricornutum CCAP 1055/1***;** 56, *Ectocarpus siliculosus***;** 57, *Nannochloropsis gaditana CCMP526***;** 58, *Emiliania huxleyi CCMP1516***;** 59, *Stylonychia lemnae***;** 60, *Oxytricha trifallax*; 61, *Plasmodiophora brassicae***; Archaea**: 62, *Candidatus methanoplasma termitum***;** 63, *Methanobacterium paludis***;** 64, *Methanocella conradii***;** 65, *Halapricum salinum***;** 66, *Methanobacterium formicicum***;** 67, *Haloterrigena limicola***; Bacteria**: 68, *Pleomorphomonas koreensis***;** 69, *Rhodopseudomonas palustris***;** 70, *Rhodomicrobium udaipurense***;** 71, *Pleomorphomonas oryzae***;** 72, *Methyloceanibacter caenitepidi***;** 73, *Halocynthiibacter namhaensis***;** 74, *Erythrobacter gangjinensis***;** 75, *Hyphomonas jannaschiana***;** 76, *Ruegeria pomeroyi*; 77, *Celeribacter baekdonensis***;** 78, *Parvibaculum lavamentivorans***;** 79, *Leisingera caerulea***;** 80, *Rubrivivax gelatinosus*; 81, *Thauera phenylacetica*; 82, *Nitrosospira briensis***;** 83, *Paludibacterium yongneupense*; 84, *Ralstonia pickettii*: 85, *Nitrosomonas eutropha*; 86, *Caldimonas manganoxidans*; 87, *Azoarcus toluclasticus*; 88, *Rhodoferax ferrireducens*; 89, *Burkholderiales bacterium GJ-E10*; 90, *Gallionella capsiferriformans*; 91, *Candidatus Thiomargarita nelsonii*; 92, *Thioalkalivibrio thiocyanodenitrificans*; 93, *Methylomarinum vadi*; 94, *Methylomicrobium buryatense*; 95, *Alcanivorax pacificus*; 96, *Simiduia agarivorans*; 97, *Porticoccus hydrocarbonoclasticus*; 98, *Oleispira antarctica*; 99, *Zooshikella ganghwensis*; 100, *Paraglaciecola psychrophila*; 101, *Hahella ganghwensis*; 102, *Saccharophagus degradans*; 103, *Azotobacter chroococcum*; 104, *Endozoicomonas numazuensis*; 105, *Colwellia psychrerythraea***;** 106, *Sedimenticola selenatireducens*; 107, *Pseudomonas alcaligenes*; 108, *Marinobacterium jannaschii*; 109, *Methylococcaceae bacterium 73a*; 134, *Nitrococcus mobilis Nb-231*; 110, *Desulfohalobium retbaense*; 111, *Desulfonatronovibrio magnus*; 112, *Desulfococcus oleovorans*; 113, *Geoalkalibacter ferrihydriticus*; 114, *Campylobacter curvus*; 115, *Mariprofundus ferrooxydans*; 116, *Tetrasphaera australiensis Ben110*; 117, *Streptomyces fradiae*; 118, *Streptomyces viridochromogenes DSM 40736*; 119, *Gordonibacter pamelaeae*; 120, *Sporolactobacillus vineae*; 121, *Desulfitobacterium hafniense*; 122, *Pelosinus fermentans JBW45*; 123, *Paenibacillus polymyxa*; 124, *Finegoldia magna*; 125, *Peptococcaceae bacterium CEB3*; 126, *Criblamydia sequanensis CRIB-18*; 127, *Sphaerobacter thermophilus DSM 20745*; 128, *Ktedonobacter racemifer DSM 44963*; 129, *Synechococcus sp*. *WH 5701*; 130, *Cyanobium gracile PCC 6307*; 131, *Nitrospira defluvii*; 132, *Gemmatimonas aurantiaca*; 133, *Bryobacter aggregatus*. Details of taxonomy and database accession numbers are given in S1 Table. The trees with the highest log likelihood inferred from a maximum likelihood analysis by MEGA6, as described in the Methods section, are shown. Numbers at the nodes reflect the percentage of 1000 replicate bootstrap trees (only values of >70 and for nodes of important branches are presented). Red circles, animal isoforms; dark blue circles, fungal isoforms; green circles, green algal isoforms; dark red circles, red algal isoforms; light blue circles, SAR; dark grey circle, amoebozoa; light gray circle, excavata; yellow circles, bacteria; and pink circles, archaea.

**Fig 4:**
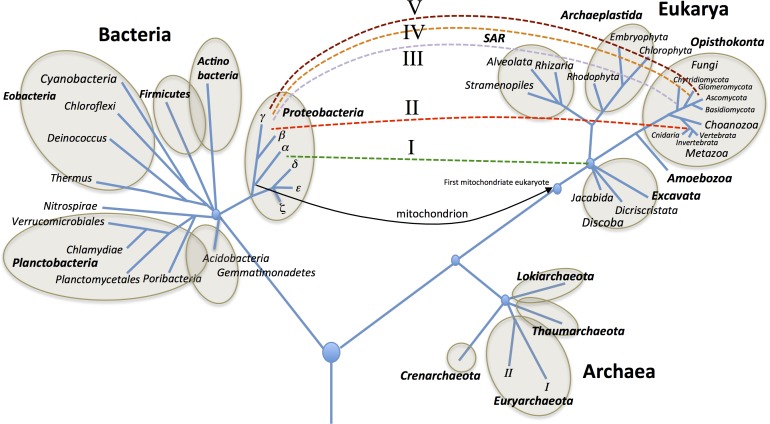
Suggested horizontal gene transfer events between bacteria and eukaryotes (dashed lines labelled I-V) for *AS3MT* throughout evolution. Schematic tree of life originally based on Woese [90] and updated by Forterre [91].

Our findings that AS3MT homologs in different species have a varied evolutionary origin could possibly be explained by assuming that organisms included in our study have differential rates of evolution, which could lead to an artifactual phylogenetic tree. Indeed, genes of the red alga *Cyanidioschyzon merolae* have been reported to be difficult to place correctly in phylogenetic trees for this reason [47]. Thus, we evaluated whether the unusual phylogeny of AS3MT was specific for this protein in the taxa investigated. To this end, we evaluated in the same taxa the evolutionary relationships between two unrelated proteins that transport potent inorganic elements, namely the calcium ATPase pump SERCA2 and the copper ATPase pump ATP7A (Fig 3; S5 and S6 Figs). In the phylogenetic trees based on these proteins, the species within each major phylum clustered together, with only one exception; the archaea clustered with bacteria in the tree based on ATP7A (no archaea species were identified carrying SERCA2 orthologs). Moreover, in the trees based on SERCA2 and ATP7A, the animal proteins were most closely related to those of fungi and most distantly related to those of bacteria (Fig 3), similar to their general distribution in the tree of life ([48–51]; Fig 4). These findings provide evidence that the unexpected phylogenetic relationships observed among AS3MT homologs are specific for this protein, and not the result of a general anomaly of evolution in the species investigated.

An alternative model for the phylogenetic pattern of the AS3MT tree could be that loss of *AS3MT* has occurred in different clades of eukaryotic lineages. If this had been the case, the eukaryotic lineages would be expected to cluster together, but with certain taxonomic groups missing. However, the patchy distribution of eukaryotic AS3MT homologs between groups of bacteria in the tree is not compatible with such a model. A further possibility is that in an arsenic-rich environment, there is a high selection pressure for mutations in any methyltransferase gene that could result in a new protein with arsenic-methylating properties. We therefore examined species that are not closely related, but have known arsenic-methylating phenotypes (*H*. *sapiens* [13], *Xenopus tropicalis* [52], the green alga *Chlamydomonas reinhardtii* [53], the red alga *Cyanidioschyzon merolae* [54], and the alpha proteobacterium *Rhodopseudomonas palustris* [55]), to establish if there were other proteins similar to AS3MT within each species, and further, to determine if these proteins showed similarities between the species. The proteins with the highest level of amino acid sequence identity to AS3MT differed between the species analysed and showed low identities and low coverage of the human AS3MT protein sequence (S3 Table); thus, numerous mutational events would be necessary to create the amino acid signature characteristic of AS3MT. We therefore consider the possibility that mutations in any methyltransferase gene gave rise to the unexpected phylogeny of AS3MT homologs rather unlikely.

### AS3MT evolution shows signs of horizontal gene transfer

To examine the robustness of our findings for the phylogeny of AS3MT, we first evaluated constrained versus unconstrained AS3MT trees constructed by RAxML followed by a Kishino-Hasegawa test. We evaluated the effects of forcing both fungi and animals to form monophyletic clusters in constrained trees. We compared the consensus MrBayes tree to the constrained and unconstrained trees from RAxML and found for both fungi and animals that the constrained trees resulted in significantly inaccurate (i.e. lower likelihood) trees compared to the unconstrained trees (S2 Table). To further test the robustness of our phylogenetic analysis, we also analysed trees based only on species in group I or group II, and the sequences in each group formed very similar branching, as shown in Fig 2 (S4 Fig). Taken together, the origin of AS3MT seems to have evolved via an unconventional route.

Based on the anomalous phylogenetic results for AS3MT, we speculated that the apparently closer relationship of animal AS3MTs to bacterial homologs rather than to other eukaryotic phyla reflects the occurrence of a HGT event between bacteria and animals. Animal and bacterial branches formed sister groups in the phylogenetic tree suggesting an early origin of HGT (Fig 2). In contrast, the unexpected phylogenetic clustering of AS3MT homologs from fungi and *H*. *magnipapillata* within bacterial clades could be explained by more recent events of HGT between bacteria and fungi as well as between bacteria and hydra.

Genomic signatures of more recent events of HGT, include the absence of introns and deviation in GC content compared with other protein-coding genes in the genome [56]. To investigate this possibility we analysed genomic sequences of *AS3MT* homologs in organisms potentially affected by HGT of *AS3MT* (S3 Table). For fungi, all species in the large ascomycete cluster have introns (typically *n* = 4) in the *AS3MT* homolog, but several of the fungal species elsewhere in the tree do not (*Serendipita vermifera*, *Trichosporon oleaginosus*, and *S*. *punctatus*) (S3 Table). The cnidarian *H*. *magnipapillata* also lacks introns in *AS3MT*. Still, for *H*. *magnipapillata* and the three fungal species that lack introns in *AS3MT*, other genes in close vicinity to *AS3MT* do carry introns, supporting the notion that these sequences are eukaryotic in origin and do not represent bacterial artefacts that occurred during extraction (S3 Table). The lack of introns in *AS3MT* in some species is in agreement with what one would expect if *AS3MT* had indeed been transferred between species and thus strengthens the argument that some species underwent HGT of *AS3MT*. Nevertheless, one should be cautious not to over-interpret these results, since the absence of introns is not always a reliable indicator of divergences that occurred long ago [57,58]. The *AS3MT* locus shows no significant differences in GC% compared with other protein-coding genes, for any of the species examined. We did not calculate the codon usage of the *AS3MT* homologs and compare it with that of highly expressed genes in the species, because we do not know which genes are highly expressed in most of the species thought to have undergone HGT of *AS3MT*.

### Correlation between arsenic methylation in animals and the presence of AS3MT

*In vitro* cell studies have shown a correlation between an arsenic-methylating phenotype and *AS3MT* genotype [59,60]. *In vivo* studies have shown that humans methylate arsenic to appreciable amounts of MMA [12] (Fig 1A), in contrast to most other mammals. Mice and dogs can methylate arsenic to DMA very efficiently, resulting in fast excretion of the resulting DMA in urine and low retention of arsenic in tissues [13]. Rats also efficiently methylate arsenic, but they retain most of the DMA in their erythrocytes, resulting in slow excretion in the urine [61]. By contrast, the marmoset monkey, chimpanzee, and guinea pig lack the ability to methylate inorganic arsenic [62–65].

As the next step of our analysis, we investigated vertebrate species for which the ability to methylate arsenic has been tested and published, and tried to correlate this phenotype with the absence or presence of putative *AS3MT* genes. Thus, we compared the sequence of human AS3MT protein (375 amino acids) with that of methyltransferase-like proteins in other species that can methylate arsenic, including rat, rhesus monkey, western clawed frog, rabbit (two isoforms), Chinese hamster (two isoforms), mouse, dog, and zebrafish, as well as two species that cannot methylate arsenic, namely the marmoset monkey and the chimpanzee (three predicted isoforms). To identify remotely related AS3MT variants in these species, BLAST searches were made with a low threshold (expect value of <e^−1^). AS3MT homologs identified in this search are listed in S4 Table.

We did not find any AS3MT sequences (protein or nucleotide sequences) in the NCBI database for guinea pig, consistent with the observation that this species cannot methylate arsenic [65]. The AS3MT sequences from all other species contained the conserved methyltransferase domain present in human AS3MT (Fig 1B). AS3MT sequences from all species showed significant alignment with human AS3MT (S7 Fig; S5 and S6 Tables). The coverage was high for all species except for marmoset, in which the coverage was only 40% and only the middle part of the marmoset protein aligned with human AS3MT, suggesting degeneration of the marmoset AS3MT gene. No such differences were seen for the other non-arsenic-methylating species, chimpanzee, for which the length of the three different predicted isoforms ranged between 364 and 377 amino acids.

Chimpanzees have three predicted AS3MT isoforms, but seem to lack the ability to methylate arsenic. It has been suggested that the lack of arsenic methylation in chimpanzee is due to a deletion in the *AS3MT* gene, resulting in a predicted 205 amino acid product of *AS3MT* that would be catalytically inactive [33]. However, since the predicted AS3MT in chimpanzee in protein databases (Uniprot and NCBI protein) is very similar in length to human AS3MT, the underlying cause for the null methylation capacity must lie elsewhere. Absence of a match between the presence of AS3MT homologs and arsenic methylation ability could be caused by loss-of-function mutations. One of the three isoforms of chimpanzee AS3MT (isoform 3; accession no. XP_016774748) lacked the cysteine at positions 32, which is necessary for the first step of arsenic methylation, but not for the second [66] and had N- and C-terminal sequences with no homology to any other AS3MT protein (S7 Fig). However, two other isoforms differed very little (by four to six amino acid residues) from human AS3MT (S7 Fig). In the central methyltransferase domain, chimpanzee sequences did not have glutamic acid (E) at position 141, in contrast to the human sequence. However, E141 is not conserved in non-mammalian AS3MTs, such as the algae *Ostreococcus* and *Chlamydomonas*, which to some extent methylate arsenic [67,68], and therefore is probably not essential for arsenic methylation capacity. Additional variant positions were identified in the N- (L4) and C-terminal domains, but these residues are not conserved in AS3MTs. Further experimental data are needed to determine whether these isoforms are actually expressed in chimpanzee and, if so, result in functional protein.

This comparative analysis showed that, with the exception of chimpanzee, animal species that do not methylate arsenic either i) lack *AS3MT*, or ii) harbour different amino acid sequences or deletions in conserved domains. These findings support the notion that AS3MT is essential for arsenic methylation capacity in nature.

## Discussion

Bacteria and archaea often adapt through HGT [69] and HGT is observed widely in eukaryotes, although here its role in adaptation is less clear. HGT has been suggested as a mechanism to explain the widespread occurrence of *arsM* (the bacterial homolog of *AS3MT*) in bacteria, based on the observation that the *arsM* gene is widespread in phylogenetically diverse bacteria [70] (Fig 2). In our study, archaea AS3MT homologs were represented in both major phylogenetic groups, suggesting that HGT of *AS3MT* had occurred between archaea and bacteria as well as between prokaryotes and eukaryotes. Recent analyses indicate that eukaryotic genomes have acquired numerous genes via HGT from prokaryotes and other lineages [71,72]. For the species in the AS3MT tree, this phenomenon has been shown for *A*. *queenslandica* [73], the cnidarians *N*. *vectensis* [74,75] and *H*. *magnipapillata* [76], which cluster with different groups of bacteria in the AS3MT tree, as well as the excavates *Naegleria gruberi* [77] and *Trichomonas vaginalis* [78], the haptophyte *E*. *huxleyi* [79], the diatom *P*. *tricornutum* [80], the red alga *Galdieria sulphuraria* [5], and the green alga *Bathycoccus prasinos* [81]. Thus, genes acquired by HGT may have contributed more to the adaptive evolution of eukaryotes than previously assumed [56,69].

The red alga *G*. *sulphuraria* can survive in high-temperature, arsenic-rich volcanic environments [5], and the presence in the *G*. *sulphuraria* genome of a gene encoding a protein with a very similar amino acid sequence to the arsenic pump ArsB in thermophilic bacteria was suggested to have resulted from HGT. The importance of *AS3MT* in *G*. *sulphuraria* is indicated by the presence of two isoforms of AS3MT, whereas most other species have only one isoform (Fig 2). However, the two AS3MT isoforms of *G*. *sulphuraria* are closely related and we therefore believe that they originated from the same HGT event. By contrast, we found indications that bacterial AS3MT isoforms could have originated from separate HGT events. Both *Desulfitobacterium hafniense* and *Sedimenticola selenatireducens* contain two isoforms of AS3MT, and in each species one isoform clusters with group I, and the other with group II.

Our comparative analysis in animals showed that the AS3MT sequence correlates with the arsenic methylating phenotype. We speculate that in a habitat with low arsenic, species like marmoset and chimpanzee have gained mutations that resulted in non-functional AS3MT, without effect on reproduction and survival. On the other hand, as humans to a larger extent have encountered an arsenic-contaminated environment during migration and expansion throughout the world, the AS3MT function has been retained. However, only few of the species that represent early branches of the animal phylogenetic tree have been analysed for arsenic methylation capacity and further functional evidence for the arsenic-methylating capacity of the proteins investigated here are warranted in future studies. It is therefore premature to speculate as to which species of animals received *AS3MT* via HGT. One candidate, however, is a close ancestor of the cnidarian *N*. *vectensis*. Cnidarians have well-established associations with bacteria [82], which have been visualized microscopically as epibionts and endosymbionts in two species of freshwater hydra [83]. This close relationship between bacteria and eukaryotic cells lacking an external physical barrier lends itself to direct host–microbe interactions. A study of the *H*. *magnipapillata* genome by Chapman et al. [76] (see their Supplemental material, Table 32) listed the *AS3MT* homolog among candidate genes potentially subjected to HGT. Our analysis strongly supports this hypothesis.

Work by Martin et al. [84] demonstrated a massive flow of genes from the genome of cyanobacteria to the nuclei of plant cells, in which the cyanobacteria exist as endosymbionts, and more recent findings showed that the endosymbiotic ancestors of mitochondria and chloroplasts brought into the eukaryotic plant and algal lineage a genome-sized sample of genes from the proteobacterial and cyanobacterial pan-genomes [85]. Endosymbiotic gene transfers occur after a unique symbiosis event and are associated with thousands of genes being transferred, In contrast, *AS3MT* acquisition is likely to have resulted from HGT, which is a more or less frequent process, in which most likely one, or a few, genes are transferred at a time. This notion is supported by the finding that i) HGT of *AS3MT* occurred on multiple occasions during evolution in widely different groups of eukaryotes, and ii) the phylogenies of two other important proteins that interact with inorganic elements, i.e., SERCA2 and ATP7A, are consistent with the generally accepted tree of life.

Based on our results and the results of others, we speculate that at least five HGT events have occurred between bacteria and eukaryotes (Fig 4); specifically, HGT has occurred: I) from bacteria to an early eukaryote and II) from bacteria to an early ancestor of the cnidarian *H*. *magnipapillata*. Further, HGT has occurred three times in fungi, namely III) from bacteria to chytridiomycota, IV) from bacteria to glomeromycota, and V) from bacteria to the branch leading to ascomycota and basidiomycota.

A striking feature of the phylogenetic tree of AS3MT protein sequences and our bioinformatics analysis is that among Viridiplantae, *AS3MT* is present in green algae (Chlorophyta), whereas land plants (Streptophyta), including mosses, lack *AS3MT*, with one exception, the charophyte *Klebsormidium flaccidum*, which represents an alga that adapted to terrestrial environments [86]. This finding indicates that *AS3MT* disappeared from land plants when they diverged from green algae, after colonization of land. The lack of AS3MT homologs in plants is surprising, given that many plants are exposed to inorganic arsenic in soil and water. Varying amounts of DMA, which likely originates from the soil, have been detected in plants, e.g. *Oryza sativa* (rice) [87]. Therefore, plants have likely developed other detoxifying processes not involving AS3MT. Research on rice and *Arabidopsis* has shown that arsenic bound to phytochelatin is efficiently sequestered to vacuoles by ABC transporters [3,88], which could represent an alternative system of detoxification. Insects also lack *AS3MT* and we suspect that, similar to plants, insects have developed alternative systems for detoxifying arsenic.

## Conclusion

Our investigation of the phylogenetics of *AS3MT* provides a novel perspective on the development of tolerance to a toxicant that occurs widely in nature. Phylogenetic analysis placed different animal and fungal AS3MTs in a prokaryotic clade with strong statistical support, consistent with HGT from multiple prokaryotic phyla. The importance of HGT for adaptation of bacteria and archaea is well established, but adaptation of resistance to a toxic environment by gene transfer from prokaryotes to eukaryotes is unexpected, and provides insight into how the environment shapes evolution. Whether HGT is a common phenomenon for acquiring detoxifying enzymes in nature remains an open question for future research. Our comparative analysis in animals revealed a close relationship between the presence of the AS3MT sequence and the capacity to methylate arsenic. The loss of arsenic methylation in certain animals likely resulted from subtle or large gene rearrangements, and may indicate that these species developed in environments with low levels of arsenic.

## Supporting information

S1 Fig Phylogenetic analysis of AS3MT proteins from animal, fungal, green and red algal, and other eukaryotic lineages, as well as bacterial and archaeal lineages, demonstrates the presence of AS3MT in different kingdoms. The tree with the highest log likelihood (-27427.9158) inferred from a maximum likelihood analysis using MEGA, as described in the Methods section, is shown. The tree shows branch lengths measured in number of substitutions per site. Numbers at the nodes reflect the percentage of 1000 replicate bootstrap trees (only values of >70 for important nodes are presented), which resolve the clade at the endpoints of that branch. AS3MT is phylogenetically split into two groups: one major group (I) divided into one subgroup of bacteria, SAR (Stramenopiles, Alveolata, Rhizaria; represented by Phatr1, Thaps1, Ects1, Emihu1), and animals; and one major group (II) of bacteria, archaea, ascomycote and basidiomycote fungi, and *Hydra magnipapillata* (Hydma1-2). The abbreviated species names and the database accession numbers are given in S1 Table. Red circles, animal isoforms; dark blue circles, fungal isoforms; green circles, green algal isoforms; dark red circles, red algal isoforms; light blue circles, SAR; dark grey circle, amoebozoa; light gray circles, excavata; yellow circles, bacteria; and pink circles, archaea. Examples of clustering of phylogenetically similar bacteria are shown with different frames: a = proteobacteria alpha in orange frames; g = proteobacteria gamma in gray frames. Scale bar, 0.2 amino acid substitutions per site.
(PDF)

S2 Fig Phylogenetic analysis of the AS3MT central domain (defined in ref. [34]) shows similar clustering as for the whole AS3MT protein in Fig 2, main text. (PDF)

S3 Fig Phylogenetic analysis of the AS3MT N-terminal domain (defined in ref. [34]) shows similar clustering as for the whole AS3MT protein in Fig 2, main text. (PDF)

S4 Fig **Phylogenetic analysis of AS3MT including only species in A) group I, and B) group II (as grouped in the AS3MT tree in Fig 2, main text) shows similar clustering of species within each group as within the tree containing both groups in Fig 2.** AS3MT is widespread in different kingdoms, and in group I a close origin of AS3MT is found in animals, SAR (Phatr1, Thaps1, Ects1; Emihu1), and one large group of phylogenetically diverse bacteria. The trees shown were derived by Bayesian inference using MrBayes, as described in Experimental Procedures.
(PDF)

S5 Fig Phylogenetic analysis of the calcium transporter SERCA2 for the same species as those evaluated for AS3MT in Fig 2 (main text). SERCA2 is widespread in different kingdoms, but is absent in archaea. The tree with the highest log likelihood (-45266.0271), inferred from a maximum likelihood analysis as described in Experimental Procedures, is shown. The tree is drawn to scale, with branch lengths measured in number of substitutions per site. The analysis involved 92 amino acid sequences. There were a total of 465 amino acid positions in the final dataset. Numbers at the nodes reflect the percentage of 1000 replicate bootstrap trees (only values of >70 are presented), which resolve the clade at the endpoints of that branch. The abbreviated species names and the database accession numbers are explained in S1 Table. Red circles, animal isoforms; dark blue circles, fungal isoforms; green circles, green algal isoforms; dark red circles, red algal isoforms; light blue circles, SAR; dark grey circle, amoebozoa; light gray circle, excavata; and yellow circles, bacteria. Scale bar, 0.2 amino acid substitutions per site.
(PDF)

S6 Fig Phylogenetic analysis of the copper transporter ATP7A for the same species as those evaluated for AS3MT in Fig 2 (main text). ATP7A is widespread in different kingdoms. The tree with the highest log likelihood (-73039.0893), inferred from a maximum likelihood analysis as described in Experimental Procedures, is shown. The tree is drawn to scale, with branch lengths measured in number of substitutions per site. The analysis involved 113 amino acid sequences. There were a total of 543 amino acid positions in the final dataset. The tree shows branch lengths measured in number of amino acid substitutions per site. Numbers at the nodes reflect the percentage of 1000 replicate bootstrap trees (only values of >70 are presented), which resolve the clade at the endpoints of that branch. The abbreviated species names and the database accession numbers are explained in S1 Table. Red circles, animal isoforms; dark blue circles, fungal isoforms; green circles, green algal isoforms; dark red circles, red algal isoforms; light blue circles, SAR; dark grey circle, amoebozoa; light gray circle, excavata; yellow circles, bacteria; and pink circles, archaea. Scale bar, 0.2 amino acid substitutions per site.
(PDF)

S7 Fig Alignment of AS3MT protein sequences for primates. The alignment contains species that can methylate arsenic: human (*Homo sapiens*) and rhesus monkey (*Macaca mulatta*), as well as two species that have been shown not to methylate arsenic (marked in red text), namely chimpanzee (*Pan troglodytes*, three isoforms) and marmoset monkey (*Callithrix jacchus*). The chimpanzee and marmoset sequences both differ from human AS3MT at one amino acid residue in the methyltransferase domain (E141; black triangle), and three amino acids outside of the methyltransferase domain (blue triangles). Since protein sequences were automatically predicted from gene sequences for several species, we manually curated amino acids that differed between human AS3MT and other species. The amino acids predicted did not differ from manually curated amino acids. The start and end of the central methyltransferase domain (as defined by Ajees and Rosen (34) are shown with thick gray horizontal arrows. Conserved cysteine residues (16; 66) are shown with red arrows. The non-synonymous human AS3MT SNP rs11191439 (methionine-threonine exchange at amino acid position 287) is shown with a green arrow. Amino acid residues that are conserved in all species in the phylogenetic tree (Fig 2) are marked with a green asterisk.
(PDF)

S1 Table Species subjected to phylogenetic analysis and gene accession numbers. (PDF)

S2 Table Analysis of constrained versus unconstrained trees by RAxML followed by a Kishino-Hasegawa test. In constrained trees, fungi and animal proteins were forced to cluster monophyletically. Statistics of constrained and unconstrained trees were subsequently scored.
(PDF)

S3 Table Analysis of AS3MT genomic sequences. (PDF)

S4 Table Inventory of AS3MT homologs in organisms that can methylate arsenic. (PDF)

S5 Table AS3MT gene and AS3MT protein sequences for selected species with data on arsenic methylation capacity. (PDF)

S6 Table Sequence similarities between the human AS3MT protein in whole and methyltransferase domains only, and other species with data on arsenic methylation capacity. (PDF)
