# Supplementary figures and images for: AS3MT-mediated tolerance to arsenic evolved by multiple independent horizontal gene transfers from bacteria to eukaryotes
Source: PLoS One. 2017 Apr 20;12(4):e0175422. doi: 10.1371/journal.pone.0175422 (PMC5398495; doi:10.1371/journal.pone.0175422)

S1 Fig

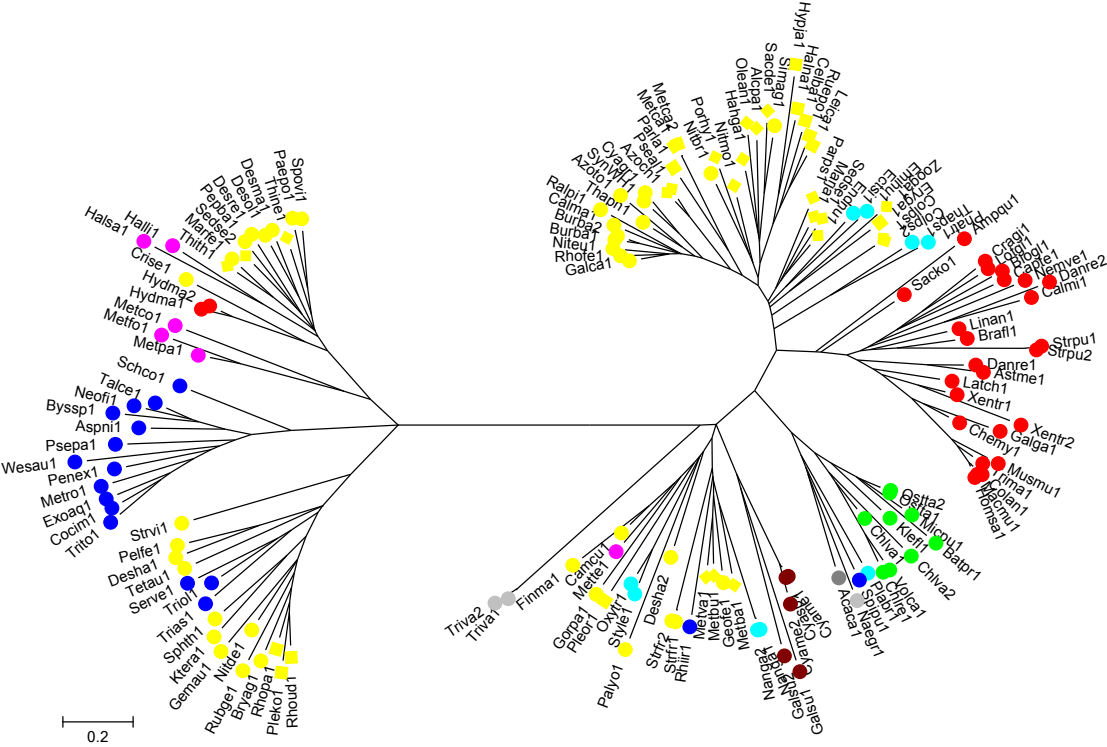

Supplement: S1 Fig — The tree with the highest log likelihood (-27427.9158) inferred from a maximum likelihood analysis using MEGA, as described in the Methods section, is shown. The tree shows branch lengths measured in number of substitutions per site. Numbers at the nodes reflect the percentage of 1000 replicate bootstrap trees (only values of >70 for important nodes are presented), which resolve the clade at the endpoints of that branch. AS3MT is phylogenetically split into two groups: one major group (I) divided into one subgroup of bacteria, SAR (Stramenopiles, Alveolata, Rhizaria; represented by Phatr1, Thaps1, Ects1, Emihu1), and animals; and one major group (II) of bacteria, archaea, ascomycote and basidiomycote fungi, and Hydra magnipapillata (Hydma1-2). The abbreviated species names and the database accession numbers are given in S1 Table. Red circles, animal isoforms; dark blue circles, fungal isoforms; green circles, green algal isoforms; dark red circles, red algal isoforms; light blue circles, SAR; dark grey circle, amoebozoa; light gray circles, excavata; yellow circles, bacteria; and pink circles, archaea. Examples of clustering of phylogenetically similar bacteria are shown with different frames: a = proteobacteria alpha in orange frames; g = proteobacteria gamma in gray frames. Scale bar, 0.2 amino acid substitutions per site. (PDF) [file pone.0175422.s001.pdf]

S3 Fig

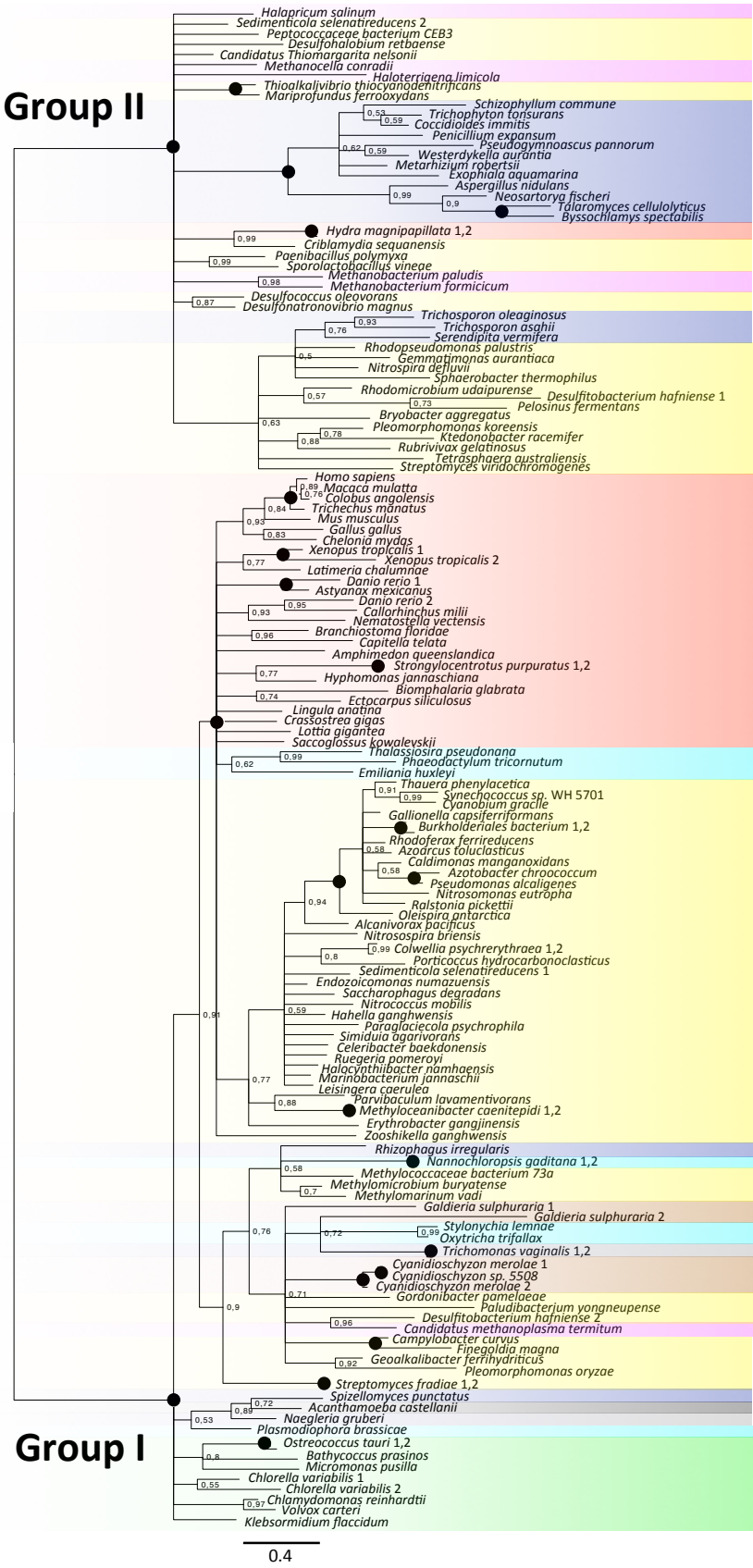

Supplement: S3 Fig — (PDF) [file pone.0175422.s003.pdf]

S4 Fig

# A

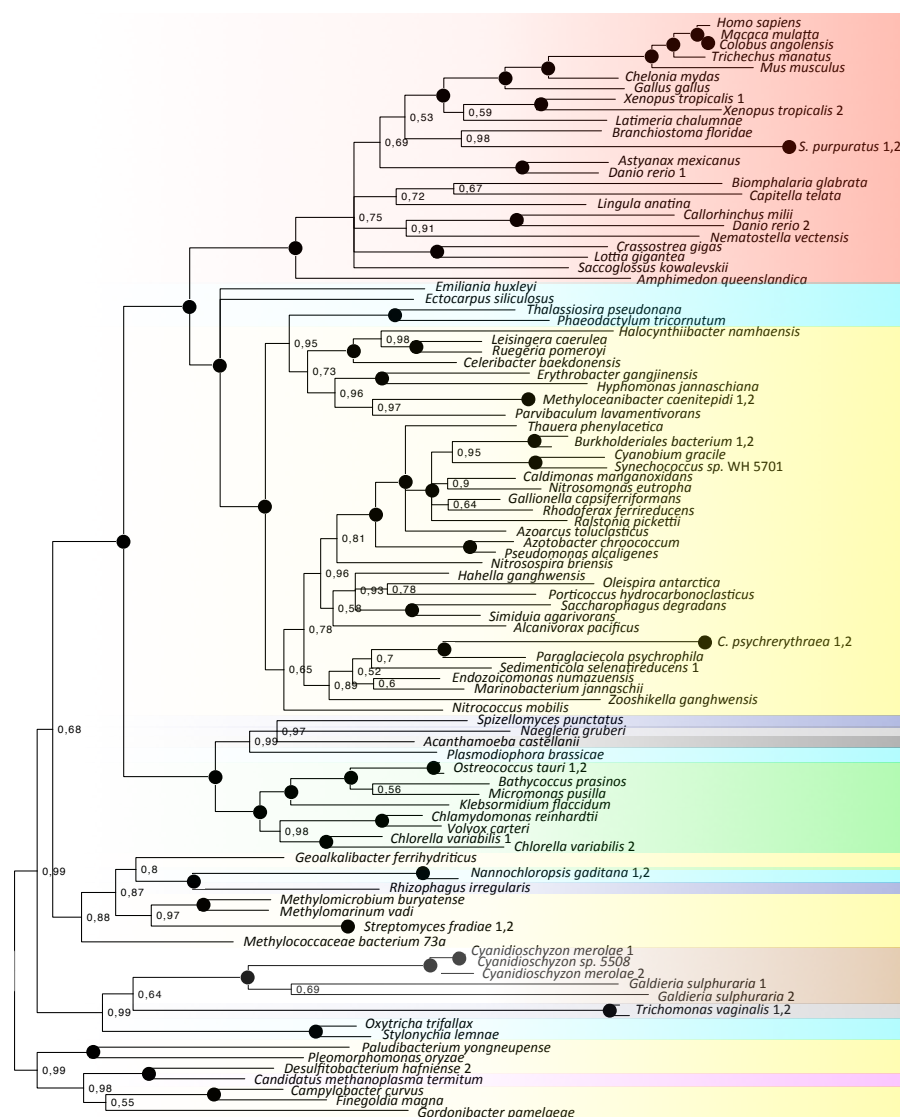

## B

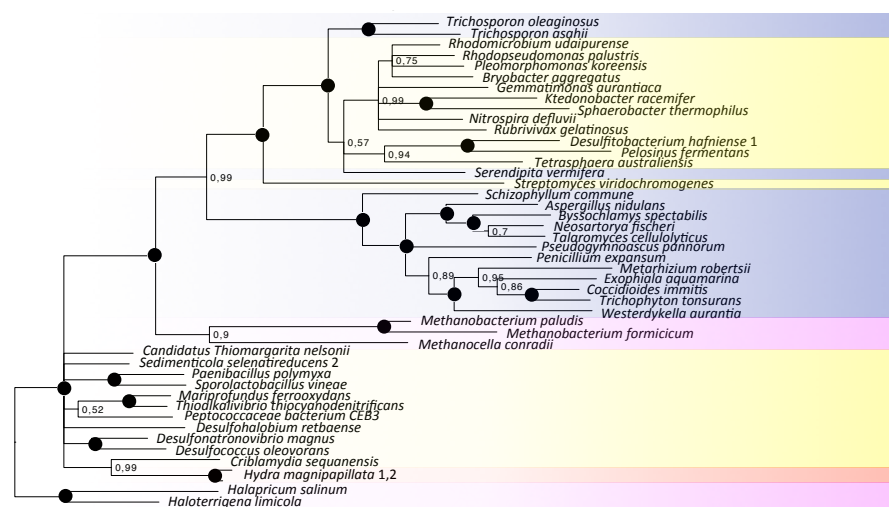

---

0.4

Supplement: S4 Fig — Phylogenetic analysis of AS3MT including only species in A) group I, and B) group II (as grouped in the AS3MT tree in Fig 2, main text) shows similar clustering of species within each group as within the tree containing both groups in Fig 2. AS3MT is widespread in different kingdoms, and in group I a close origin of AS3MT is found in animals, SAR (Phatr1, Thaps1, Ects1; Emihu1), and one large group of phylogenetically diverse bacteria. The trees shown were derived by Bayesian inference using MrBayes, as described in Experimental Procedures. (PDF) [file pone.0175422.s004.pdf]

S5 Fig

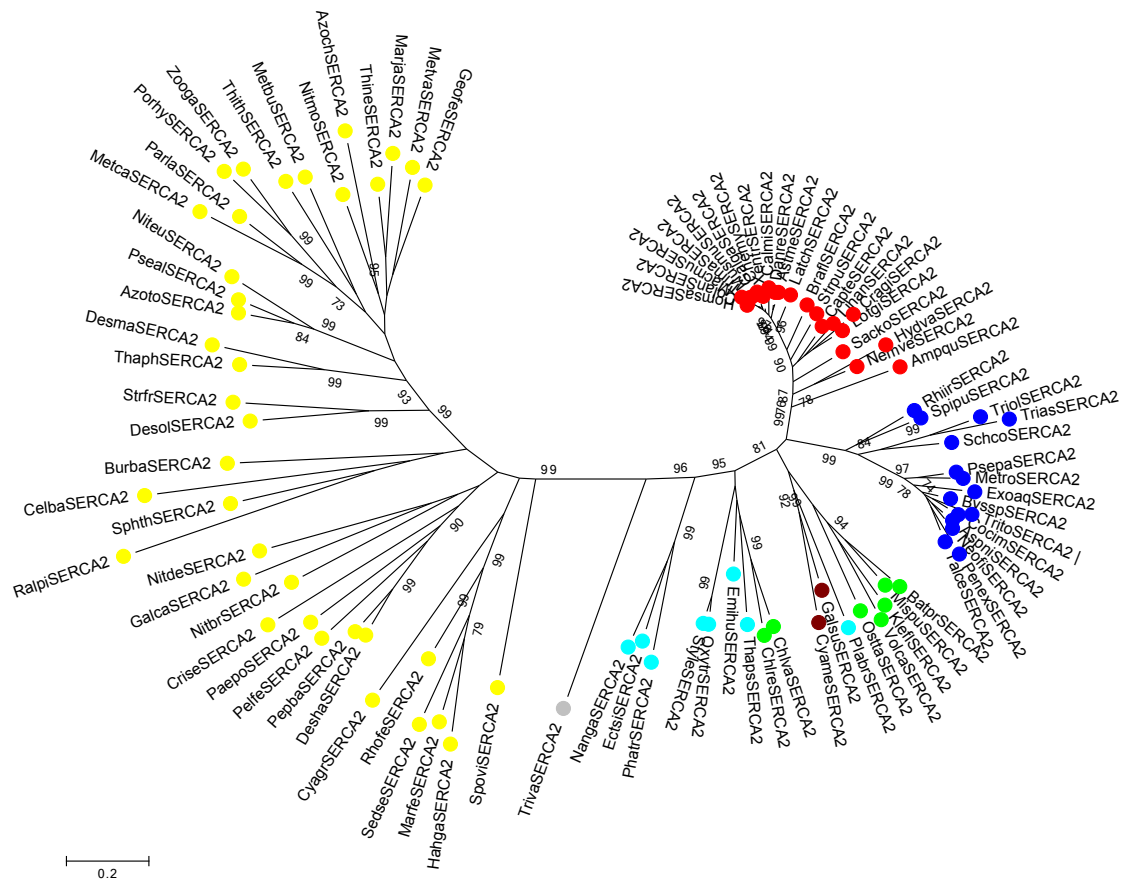

Supplement: S5 Fig — SERCA2 is widespread in different kingdoms, but is absent in archaea. The tree with the highest log likelihood (-45266.0271), inferred from a maximum likelihood analysis as described in Experimental Procedures, is shown. The tree is drawn to scale, with branch lengths measured in number of substitutions per site. The analysis involved 92 amino acid sequences. There were a total of 465 amino acid positions in the final dataset. Numbers at the nodes reflect the percentage of 1000 replicate bootstrap trees (only values of >70 are presented), which resolve the clade at the endpoints of that branch. The abbreviated species names and the database accession numbers are explained in S1 Table. Red circles, animal isoforms; dark blue circles, fungal isoforms; green circles, green algal isoforms; dark red circles, red algal isoforms; light blue circles, SAR; dark grey circle, amoebozoa; light gray circle, excavata; and yellow circles, bacteria. Scale bar, 0.2 amino acid substitutions per site. (PDF) [file pone.0175422.s005.pdf]

S6 Fig

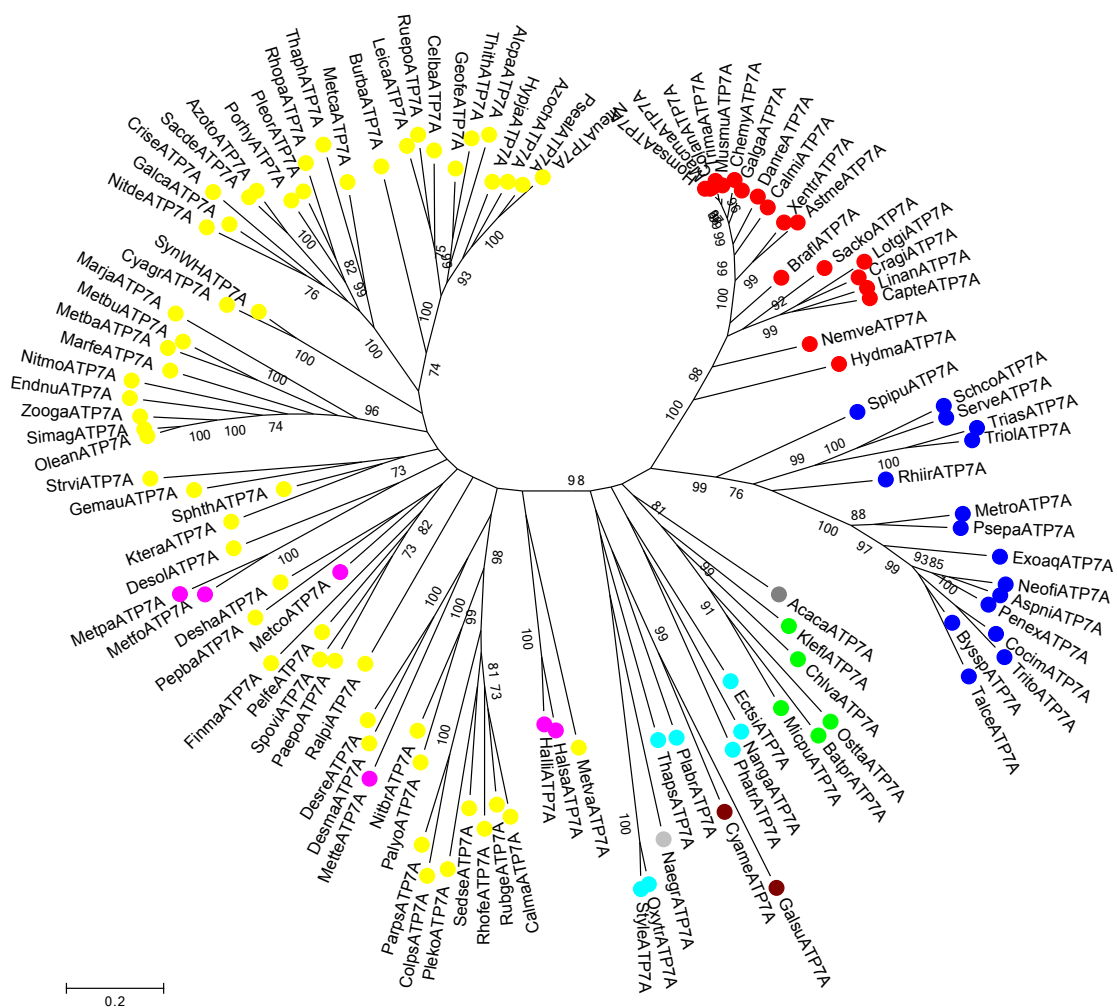

Supplement: S6 Fig — ATP7A is widespread in different kingdoms. The tree with the highest log likelihood (-73039.0893), inferred from a maximum likelihood analysis as described in Experimental Procedures, is shown. The tree is drawn to scale, with branch lengths measured in number of substitutions per site. The analysis involved 113 amino acid sequences. There were a total of 543 amino acid positions in the final dataset. The tree shows branch lengths measured in number of amino acid substitutions per site. Numbers at the nodes reflect the percentage of 1000 replicate bootstrap trees (only values of >70 are presented), which resolve the clade at the endpoints of that branch. The abbreviated species names and the database accession numbers are explained in S1 Table. Red circles, animal isoforms; dark blue circles, fungal isoforms; green circles, green algal isoforms; dark red circles, red algal isoforms; light blue circles, SAR; dark grey circle, amoebozoa; light gray circle, excavata; yellow circles, bacteria; and pink circles, archaea. Scale bar, 0.2 amino acid substitutions per site. (PDF) [file pone.0175422.s006.pdf]
