# Supplementary material for: AS3MT-mediated tolerance to arsenic evolved by multiple independent horizontal gene transfers from bacteria to eukaryotes
Source: PLoS One. 2017 Apr 20;12(4):e0175422. doi: 10.1371/journal.pone.0175422 (PMC5398495; doi:10.1371/journal.pone.0175422)
Supplement: S2 Fig — (PDF) [file pone.0175422.s002.pdf]

S2 Fig

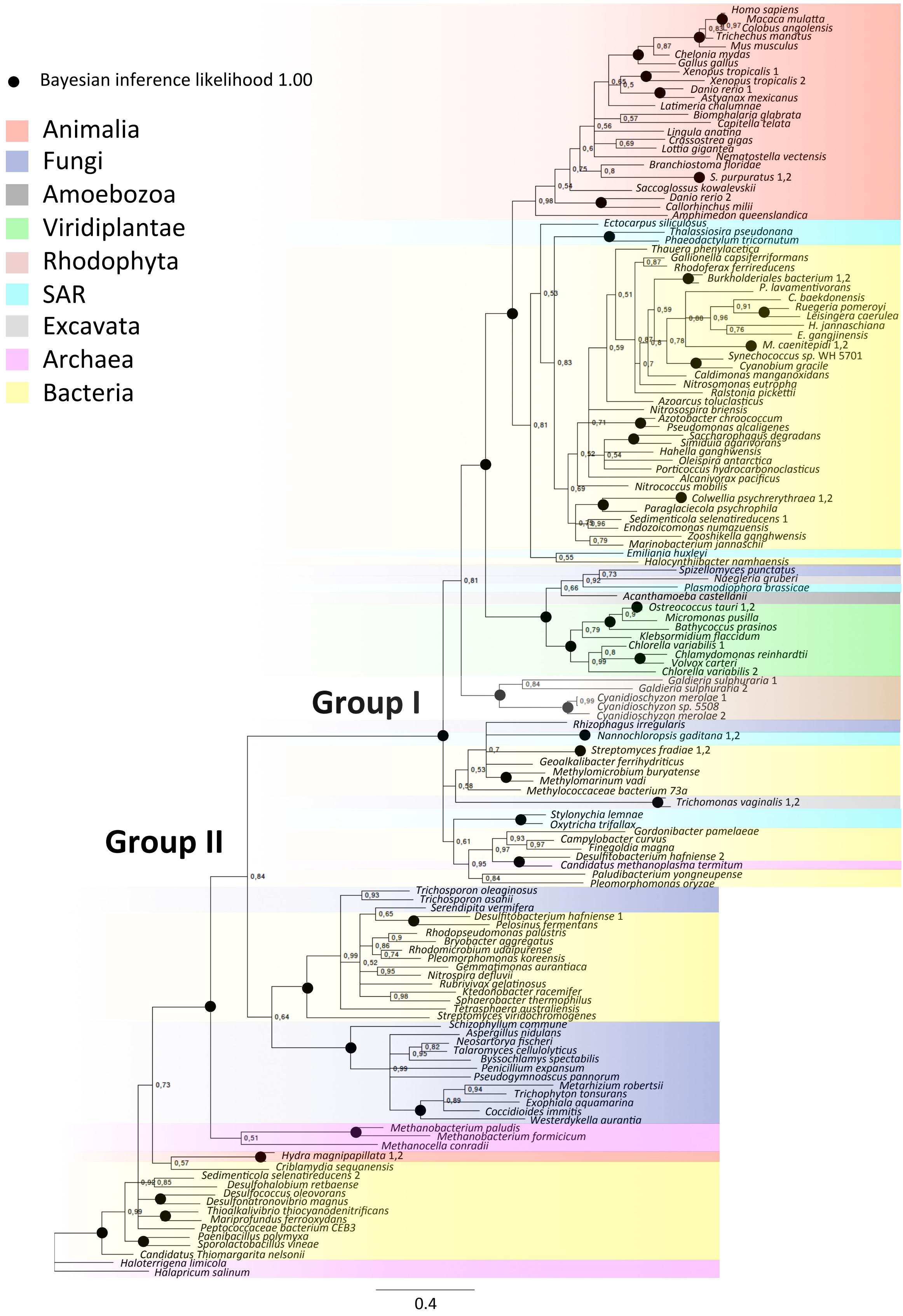
Bayesian inference likelihood 1.00
Animalia
Fungi
Amoebozoa
Viridiplantae
Rhodophyta
SAR
Excavata
Archaea
Bacteria
Group I
Group II
Homo sapiens
Macaca mulatta
Colobus angolensis
Trichechus manatus
Mus musculus
Chelonia mydas
Gallus gallus
Xenopus tropicalis 1
Xenopus tropicalis 2
Danio rerio 1
Astyanax mexicanus
Latimeria chalumnae
Biomphalaria glabrata
Capitella telata
Lingula anatina
Crassostrea gigas
Lottia gigantea
Nematostella vectensis
Branchiostoma floridae
S. purpuratus 1,2
Saccoglossus kowalevskii
Danio rerio 2
Callorhinchus milii
Amphimedon queenslandica
Ectocarpus siliculosus
Thalassiosira pseudonana
Phaeodactylum tricornutum
Thauera phenylacetica
Gallionella capsiferriformans
Rhodoferax ferrireducens
Burkholderiales bacterium 1,2
P. lavamentivorans
C. baekdonensis
Ruegeria pomeroyi
Leisingera caerulea
H. jannaschiana
E. gangjinensis
M. caenitepidi 1,2
Synechococcus sp. WH 5701
Cyanobium gracile
Caldimonas manganoxidans
Nitrosomonas eutropha
Ralstonia pickettii
Azoarcus toluclasticus
Nitrosospira briensis
Azotobacter chroococcum
Pseudomonas alcaligenes
Saccharophagus degradans
Simiduia agarivorans
Hahella ganghwensis
Oleispira antarctica
Porticoccus hydrocarbonoclasticus
Alcanivorax pacificus
Nitrococcus mobilis
Colwellia psychrerythraea 1,2
Paraglaciecola psychrophila
Sedimenticola selenatireducens 1
Endozoicomonas numazuensis
Zooshikella ganghwensis
Marinobacterium jannaschii
Emiliania huxleyi
Halocynthiibacter namhaensis
Spizellomyces punctatus
Naegleria gruberi
Plasmodiophora brassicae
Acanthamoeba castellanii
Ostreococcus tauri 1,2
Micromonas pusilla
Bathycoccus prasinos
Klebsormidium flaccidum
Chlorella variabilis 1
Chlamydomonas reinhardtii
Volvox carteri
Chlorella variabilis 2
Galdieria sulphuraria 1
Galdieria sulphuraria 2
Cyanidioschyzon merolae 1
Cyanidioschyzon sp. 5508
Cyanidioschyzon merolae 2
Rhizophagus irregularis
Nannochloropsis gaditana 1,2
Streptomyces fradiae 1,2
Geoalkalibacter ferrihydriticus
Methylomicrobium buryatense
Methylomarinum vadi
Methylococcaceae bacterium 73a
Trichomonas vaginalis 1,2
Stylonychia lemnae
Oxytricha trifallax
Gordonibacter pamelaeae
Campylobacter curvus
Finegoldia magna
Desulfitobacterium hafniense 2
Candidatus methanoplasma termitum
Paludibacterium yongneupense
Pleomorphomonas oryzae
Trichosporon oleaginosus
Trichosporon asahii
Serendipita vermifera
Desulfitobacterium hafniense 1
Pelosinus fermentans
Rhodopseudomonas palustris
Bryobacter aggregatus
Rhodomicrobium udaipurense
Pleomorphomonas koreensis
Gemmatimonas aurantiaca
Nitrospira defluvii
Rubrivivax gelatinosus
Ktedonobacter racemifer
Sphaerobacter thermophilus
Tetrasphaera australiensis
Streptomyces viridochromogenes
Schizophyllum commune
Aspergillus nidulans
Neosartorya fischeri
Talaromyces cellulolyticus
Byssochlamys spectabilis
Penicillium expansum
Pseudogymnoascus pannorum
Metarhizium robertsii
Trichophyton tonsurans
Exophiala aquamarina
Coccidioides immitis
Westerdykella aurantia
Methanobacterium paludis
Methanobacterium formicicum
Methanocella conradii
Hydra magnipapillata 1,2
Criblamydia sequanensis
Sedimenticola selenatireducens 2
Desulfohalobium retbaense
Desulfococcus oleovorans
Desulfonatronovibrio magnus
Thioalkalivibrio thiocyanodenitrificans
Mariprofundus ferrooxydans
Peptococcaceae bacterium CEB3
Paenibacillus polymyxa
Sporolactobacillus vineae
Candidatus Thiomargarita nelsonii
Haloterrigena limicola
Halapricum salinum
0.4
