# Supplementary material for: AS3MT-mediated tolerance to arsenic evolved by multiple independent horizontal gene transfers from bacteria to eukaryotes
Source: PLoS One. 2017 Apr 20;12(4):e0175422. doi: 10.1371/journal.pone.0175422 (PMC5398495; doi:10.1371/journal.pone.0175422)
Supplement: S7 Fig — The alignment contains species that can methylate arsenic: human (Homo sapiens) and rhesus monkey (Macaca mulatta), as well as two species that have been shown not to methylate arsenic (marked in red text), namely chimpanzee (Pan troglodytes, three isoforms) and marmoset monkey (Callithrix jacchus). The chimpanzee and marmoset sequences both differ from human AS3MT at one amino acid residue in the methyltransferase domain (E141; black triangle), and three amino acids outside of the methyltransferase domain (blue triangles). Since protein sequences were automatically predicted from gene sequences for several species, we manually curated amino acids that differed between human AS3MT and other species. The amino acids predicted did not differ from manually curated amino acids. The start and end of the central methyltransferase domain (as defined by Ajees and Rosen (34) are shown with thick gray horizontal arrows. Conserved cysteine residues (16; 66) are shown with red arrows. The non-synonymous human AS3MT SNP rs11191439 (methionine-threonine exchange at amino acid position 287) is shown with a green arrow. Amino acid residues that are conserved in all species in the phylogenetic tree (Fig 2) are marked with a green asterisk. (PDF) [file pone.0175422.s007.pdf]

S7 Fig

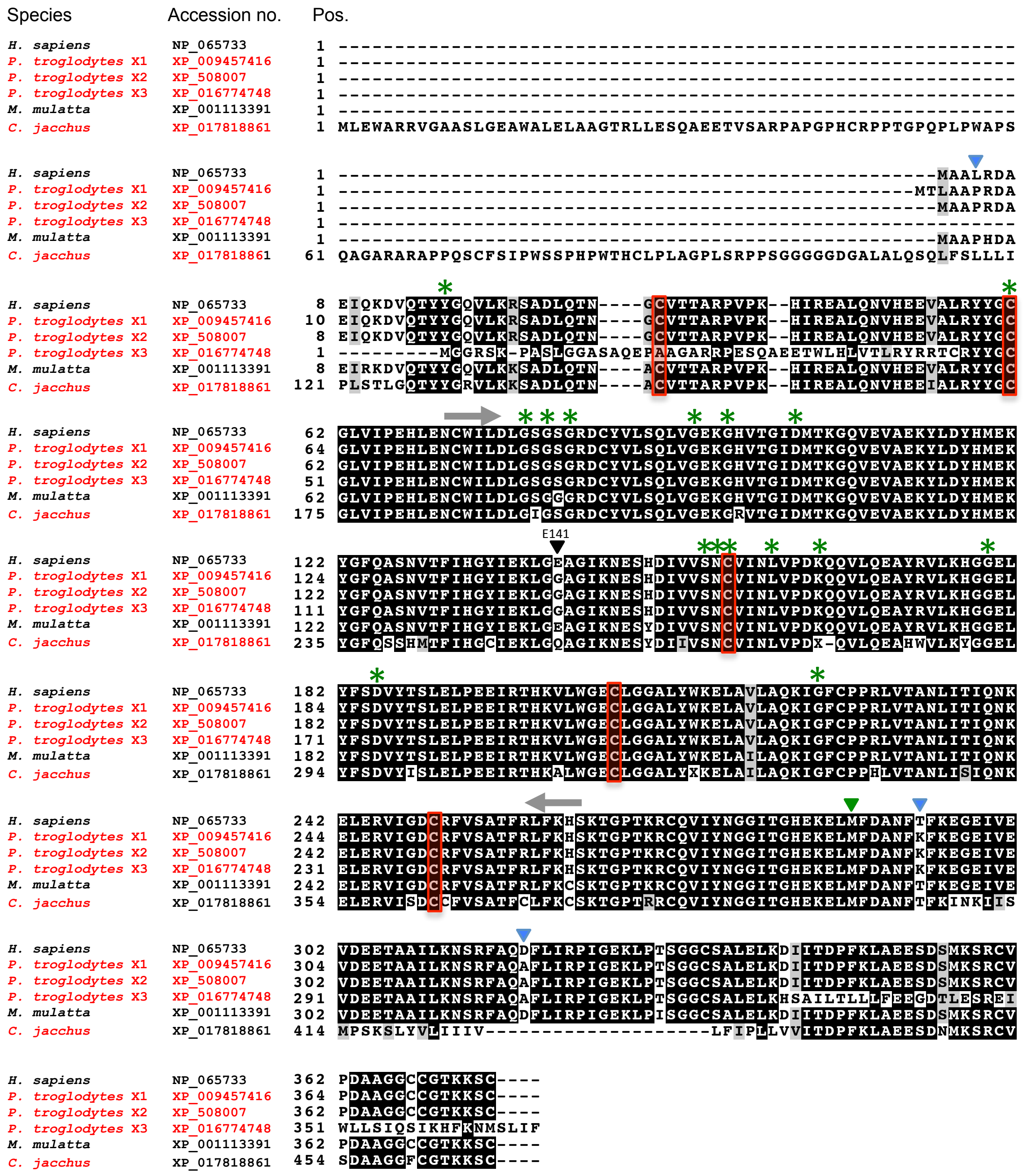
Species
Accession no.
Pos.
H. sapiens NP_065733 1 --
P. troglodytes X1 XP_009457416 1 --
P. troglodytes X2 XP_508007 1 --
P. troglodytes X3 XP_016774748 1 --
M. mulatta XP_001113391 1 --
C. jacchus XP_017818861 1 MLEWARRVGAASLGEAWALELAAGTRLLESQAEETVSARPAPGPHCRPPTGPQPLPWAPS
H. sapiens NP_065733 1 ---MAALRDA
P. troglodytes X1 XP_009457416 1 ---MTLAAPRDA
P. troglodytes X2 XP_508007 1 ---MAAPRDA
P. troglodytes X3 XP_016774748 1 --
M. mulatta XP_001113391 1 ---MAAPHDA
C. jacchus XP_017818861 61 QAGARARAPPQSCFSIPWSSPHPWTHCLPLAGPLSRPPSGGGGGDGALALQSQLFSLLLI
H. sapiens NP_065733 8 EIQKDVQTYYGQVLKRSADLQTN----GCVTTARPVPK--HIREALQNVHEEVALRYYGC
P. troglodytes X1 XP_009457416 10 EIQKDVQTYYGQVLKRSADLQTN----GCVTTARPVPK--HIREALQNVHEEVALRYYGC
P. troglodytes X2 XP_508007 8 EIQKDVQTYYGQVLKRSADLQTN----GCVTTARPVPK--HIREALQNVHEEVALRYYGC
P. troglodytes X3 XP_016774748 1 ---------MGGRSK-PASLGGASAQEPAAGARRPESQAEETWLHLVTLRYRRTCRYYGC
M. mulatta XP_001113391 8 EIRKDVQTYYGRVLKKSADLQTN----ACVTTARPVPK--HIREALQNVHEEVALRYYGC
C. jacchus XP_017818861 121 PLSTLGQTYYGRVLKKSADLQTN----ACVTTARPVPK--HIREALQNVHEEIALRYYGC
H. sapiens NP_065733 62 GLVIPEHLENCWILDLGSGSGRDCYVLSQLVGEKGHVTGIDMTKGQVEVAEKYLDYHMEK
P. troglodytes X1 XP_009457416 64 GLVIPEHLENCWILDLGSGSGRDCYVLSQLVGEKGHVTGIDMTKGQVEVAEKYLDYHMEK
P. troglodytes X2 XP_508007 62 GLVIPEHLENCWILDLGSGSGRDCYVLSQLVGEKGHVTGIDMTKGQVEVAEKYLDYHMEK
P. troglodytes X3 XP_016774748 51 GLVIPEHLENCWILDLGSGSGRDCYVLSQLVGEKGHVTGIDMTKGQVEVAEKYLDYHMEK
M. mulatta XP_001113391 62 GLVIPEHLENCWILDLGSGSGRDCYVLSQLVGEKGHVTGIDMTKGQVEVAEKYLDYHMEK
C. jacchus XP_017818861 175 GLVIPEHLENCWILDLGIGSGRDCYVLSQLVGEKGRVTGIDMTKGQVEVAEKYLDYHMEK
E141
H. sapiens NP_065733 122 YGFQASNVTFIHGYIEKLGEAGIKNESHDIVVSNCVINLVPDKQQVLQEAYRVLKHGGEL
P. troglodytes X1 XP_009457416 124 YGFQASNVTFIHGYIEKLGGAGIKNESHDIVVSNCVINLVPDKQQVLQEAYRVLKHGGEL
P. troglodytes X2 XP_508007 122 YGFQASNVTFIHGYIEKLGGAGIKNESHDIVVSNCVINLVPDKQQVLQEAYRVLKHGGEL
P. troglodytes X3 XP_016774748 111 YGFQASNVTFIHGYIEKLGGAGIKNESHDIVVSNCVINLVPDKQQVLQEAYRVLKHGGEL
M. mulatta XP_001113391 122 YGFQASNVTFIHGYIEKLGEAGIKNESYDIVVSNCVINLVPDKQQVLQEAYRVLKHGGEL
C. jacchus XP_017818861 235 YGFQSSHMTFIHGCIEKLGQAGIKNESYDIIVSNCVINLVPDX-QVLQEAHWVLKYGGEL
H. sapiens NP_065733 182 YFSDVYTSLELPEEIRTHKVLWGECLGGALYWKELAVLAQKIGFCPPRLVTANLITIQNK
P. troglodytes X1 XP_009457416 184 YFSDVYTSLELPEEIRTHKVLWGECLGGALYWKELAVLAQKIGFCPPRLVTANLITIQNK
P. troglodytes X2 XP_508007 182 YFSDVYTSLELPEEIRTHKVLWGECLGGALYWKELAVLAQKIGFCPPRLVTANLITIQNK
P. troglodytes X3 XP_016774748 171 YFSDVYTSLELPEEIRTHKVLWGECLGGALYWKELAVLAQKIGFCPPRLVTANLITIQNK
M. mulatta XP_001113391 182 YFSDVYTSLELPEEIRTHKVLWGECLGGALYWKELAILAQKIGFCPPRLVTANLITIQNK
C. jacchus XP_017818861 294 YFSDVYISLELPEEIRTHKALWGECLGGALYXKELAILAQKIGFCPPHLVTANLISIQNK
H. sapiens NP_065733 242 ELERVIGDCRFVSATFRLFKHSKTGPTKRCQVIYNGGITGHEKELMFDANFTFKEGEIVE
P. troglodytes X1 XP_009457416 244 ELERVIGDCRFVSATFRLFKHSKTGPTKRCQVIYNGGITGHEKELMFDANFKFKEGEIVE
P. troglodytes X2 XP_508007 242 ELERVIGDCRFVSATFRLFKHSKTGPTKRCQVIYNGGITGHEKELMFDANFKFKEGEIVE
P. troglodytes X3 XP_016774748 231 ELERVIGDCRFVSATFRLFKHSKTGPTKRCQVIYNGGITGHEKELMFDANFKFKEGEIVE
M. mulatta XP_001113391 242 ELERVIGDCRFVSATFRLFKCSKTGPTKRCQVIYNGGITGHEKELMFDANFTFKEGEIVE
C. jacchus XP_017818861 354 ELERVISDCCFVSATFCLFKCSKTGPTRRCQVIYNGGITGHEKELMFDANFTFKINKIIS
H. sapiens NP_065733 302 VDEETAAILKNSRFAQDFLIRPIGEKLPTSGGCSALELKDIITDPFKLAEESDSMKSRCV
P. troglodytes X1 XP_009457416 304 VDEETAAILKNSRFAQAFLIRPIGEKLPTSGGCSALELKDIITDPFKLAEESDSMKSRCV
P. troglodytes X2 XP_508007 302 VDEETAAILKNSRFAQAFLIRPIGEKLPTSGGCSALELKDIITDPFKLAEESDSMKSRCV
P. troglodytes X3 XP_016774748 291 VDEETAAILKNSRFAQAFLIRPIGEKLPTSGGCSALELKHSAILTLLLFEEGDTLESREI
M. mulatta XP_001113391 302 VDEETAAILKNSRFAQDFLIRPIGEKLPISGGCSALELKDIITDPFKLAEESDSMKSRCV
C. jacchus XP_017818861 414 MPSKSLYVLIIIV--------------------LFIPLLVVITDPFKLAEESDNMKSRCV
H. sapiens NP_065733 362 PDAAGGCCGTKKSC----
P. troglodytes X1 XP_009457416 364 PDAAGGCCGTKKSC----
P. troglodytes X2 XP_508007 362 PDAAGGCCGTKKSC----
P. troglodytes X3 XP_016774748 351 WLLSIQSIKHFKNMSLIF
M. mulatta XP_001113391 362 PDAAGGCCGTKKSC----
C. jacchus XP_017818861 454 SDAAGGFCGTKKSC----
