# Supplementary material for: AS3MT-mediated tolerance to arsenic evolved by multiple independent horizontal gene transfers from bacteria to eukaryotes
Source: PLoS One. 2017 Apr 20;12(4):e0175422. doi: 10.1371/journal.pone.0175422 (PMC5398495; doi:10.1371/journal.pone.0175422)
Supplement: S1 Table — (PDF) [file pone.0175422.s008.pdf]

S1 Table									
**Species subjected to phylogenetic analysis and gene accession numbers**									
				AS3MT homologues		***ATP7A homologues***		***SERCA2 homologues***	
Superkingdom	***Taxonomy (phy***	***Species***	***No***	***Name abbrev***	***Acc. No.***	***Name abbrev***	***Acc. No.***	***Name abbrev***	***Acc. No.***
**Opisthokonta**	**Chordata**								
	Mammalia								
		Homo sapiens	1	Homsa1	Q9HBK9	HomsaATP7A	Q04656	HomsaSERCA2	P16615
		Macaca mulatta	2	Macmu1	XP_001113391.2	MacmaATP7A	EHH30866.1	MacmuSERCA2	EHH21172.1
		Colobus angolensis palliatus	3	Colan1	XP_011812758.1	ColanATP7A	XP_011802825.1	ColanSERCA2	XP_011791730.1
		Trichechus manatus latirostris	4	Trima1	XP_004370149.1	TrimaATP7A	XP_004378951.1	TrimaSERCA2	XP_004379145.1
		Mus musculus	5	Musmu1	NP_065602.2	MusmuATP7A	Q64430.3	MusmuSERCA2	O55143.2
	Aves								
		Gallus gallus	6	Galga1	XP_421735.3	GalgaATP7A	XP_004940860.1	GalgaSERCA2	Q03669.2
	Reptilia								
		Chelonia mydas	7	Chemy1	XP_007057932.1	ChemyATP7A	XP_007060223.1	ChemySERCA2	XP_007058345.1
	Actinopterygii								
		Danio rerio	8a	Danre1	NP_001034928.1	DanreATP7A	XP_005173182.1	DanreSERCA2	XP_005161253.1
			8b	Danre2	Zgc:153372				
		Astyanax mexicanus	9	Astme1	XP_007253206.1	AstmeATP7A	XP_007248959.1	AstmeSERCA2	XP_007252855.1
	Chondrichthyes								
		Callorhinchus milii	10	Calmi1	XP_007882715.1	CalmiATP7A	XP_007892184.1	CalmiSERCA2	XP_007900428.1
	Sarcopterigii								
		Latimeria chalumnae	11	Latch1	XP_006007443.1			LatchSERCA2	XP_006005927.1
	Cephalochordata								
		Branchiostoma floridae	12	Brafl1	XP_002609029.1	BraflATP7A	XP_002611623.1	BraflSERCA2	XP_002612126.1
	Amphibia								
		Xenopus tropicalis	13a	Xentr1	NP_001135714.1	XentrATP7A	XP_002936778.2	XentrSERCA2	XP_004910568.1
			13b	Xentr2	XP_002938772.2				

	**Hemichordata**								
	Enteropneusta	*Saccoglossus kowalevskii*	14	Sacko1	XP_002736526.1	SackoATP7A	XP_006820395.1	SackoSERCA2	XP_006821107.1
	**Invertebrata**								
	**Annelida**								
	Polychaeta								
		Capitella telata	15	Capte1	ELU09208.1	CapteATP7A	ELU04312.1	CapteSERCA2	ELU08428.1
	**Mollusca**								
	Bivalvia								
		Crassostrea gigas	16	Cragi1	XP_011430074.1	CragiATP7A	XP_011442669.1	CragiSERCA2	XP_011425752.1
	Gastropoda								
		Lottia gigantea	17	Lotgi1	XP_009064258.1	LotgiATP7A	XP_009044906.1	LotgiSERCA2	XP_009051621.1
		Biomphalaria glabrata	18	Biogl1	XP_013076666.1				
	**Brachiopoda**								
	Lingulata								
		Lingula anatina	19	Linan1	XP_013410490.1	LinanATP7A	XP_013413555.1	LinanSERCA2	XP_013421498.1
	**Echinodermata**								
	Echinoidea								
		Strongylocentrotus purpuratus	20a	Strpu1	XP_784275.2	StrpuATP7A	XP_011661047.1	StrpuSERCA2	XP_011663710.1
			20b	Strup2	XP_793985.2				
	**Cnidaria**								
	Hydrozoa								
		Hydra magnipapillata	21a	Hydma1	XP_002165192.1	HydmaATP7A	XP_012564496.1	HydmaSERCA2	XP_012557152.1
			21b	Hydma2	XP_012554525.1				
	Anthozoa								
		Nematostella vectensis	22	Nemve1	XP_001635188.1	NemveATP7A	XP_001639143.1	NemveSERCA2	XP_001639528.1

	**Porifera**								
	Haplosclerida								
		Amphimedon queenslandica	23	Ampqu1	XP_011409148.1			AmpquSERCA2	XP_011403336.1
	**Fungi**								
	**Ascomycota**								
	Eurotiomycetes								
		Aspergillus nidulans FGSC A4	24	Aspni1	XP_662229.1|	AspniATP7A	XP_661228.1	AspniSERCA2	CBF81294.1
		Penicillium expansum	25	Penex1	KGO36850.1	PenexATP7A	KGO37838.1	PenexSERCA2	KGO39059.1
		Neosartorya fischeri NRRL 181	26	Neofi1	XP_001262195.1	NeofiATP7A	XP_001266778.1	NeofiSERCA2	XP_001257791.1
		Talaromyces cellulolyticus	27	Talce1	GAM41075.1	TalceATP7A	GAM41791.1	TalceSERCA2	GAM40431.1
		Byssochlamys spectabilis No. 5	28	Byssp1	GAD92999.1	BysspATP7A	GAD99045.1	BysspSERCA2	GAD96377.1
		Trichophyton tonsurans CBS 11	29	Trito1	EGD94376.1	TritoATP7A	EGE00321.1	TritoSERCA2	EGD96734.1
		Exophiala aquamarina CBS 119	30	Exoaq1	XP_013258903.1	ExoaqATP7A	XP_013259450.1	ExoaqSERCA2	XP_013265640.1
		Coccidioides immitis RS	31	Cocim1	XP_012213960.1	CocimATP7A	XP_001246821.1	CocimSERCA2	XP_001243422.2
	Leotiomycetes								
		Pseudogymnoascus pannorum	36	Psepa1	KFY38374.1	PsepaATP7A	KFY41366.1	PsepaSERCA2	KFY41655.1
		Westerdykella aurantiaca	37	Wesau1	AJG44816.1				
	Sordariomycetes								
		Metarhizium robertsii ARSEF 2	38	Metro1	XP_007826674.1	MetroATP7A	XP_007816715.1	MetroSERCA2	XP_007823469.2
	Basidiomycota								
		Trichosporon oleaginosus	32	Triol1	KLT40399.1	TriolATP7A	KLT46504.1	TriolSERCA2	KLT45999.1
		Serendipita vermifera MAFF 30	33	Serve1	KIM29307.1	ServeATP7A	KIM33903.1		
		Trichosporon asahii var. asahii	34	Trias1	EKD03641.1	TriasATP7A	EKD04276.1	TriasSERCA2	EKD03529.1
		Schizophyllum commune H4-8	35	Schco1	XP_003037640.1	SchcoATP7A	XP_003032834.1	SchcoSERCA2	XP_003035126.1

	Leotiomycetes								
		Pseudogymnoascus pannorum	36	Psepa1	KFY38374.1	PsepaATP7A	KFY41366.1	PsepaSERCA2	KFY41655.1
		Westerdykella aurantiaca	37	Wesau1	AJG44816.1				
	Sordariomycetes								
		Metarhizium robertsii ARSEF 2[illegible]	38	Metro1	XP_007826674.1	MetroATP7A	XP_007816715.1	MetroSERCA2	XP_007823469.2
	**Glomeromycota**								
	Glomeromycetes								
		Rhizophagus irregularis	39	Rhiir1	ESA08194.1	RhiirATP7A	ESA07092.1	RhiirSERCA2	ESA01669.1
	**Chytridiomycota**								
	Chytridiomycetes								
		Spizellomyces punctatus DAOM	40	Spipu1	KND00557.1	SpipuATP7A	KNC99072.1	SpipuSERCA2	KNC99029.1
**Archaeplastida**	**Chlorophyta**								
	Chlorophyceae								
		Chlamydomonas reinhardtii	41	Chlre1	AFS88933.2	ChlreATP7A	XP_001699267.1	ChlreSERCA2	XP_001700727.1
		Volvox carteri f. nagariensis	42	Volca1	XP_002954859.1	VolcaATP7A	XP_002958680.1	VolcaSERCA2	XP_002953236.1
	Trebouxiophyceae								
		Chlorella variabilis	43a	Chlva1	XP_005845903.1	ChlvaATP7A	XP_005852165.1	ChlvaSERCA2	XP_005847889.1
			43b	Chlva2	XP_005847544.1				
	Mamiellophyceae								
		Micromonas pusilla CCMP1545	44	Micpu1	XP_003064194.1	MicpuATP7A	XP_003059255.1	MispuSERCA2	XP_003055417.1
		Ostreococcus tauri	45a	Ostta1	CEG00520.1	OsttaATP7A	XP_003079479.1	OsttaSERCA2	CEF97299.1
			45b	Ostta2	XP_003083819.1				
		Bathycoccus prasinos	46	Batpr1	XP_007512671.1	BatprATP7A	XP_007514218.1	BatprSERCA2	XP_007512204.1
	**Charophyta**								

	Klebsormidiales								
		Klebsormirdium flaccidum	47	Klefl1	kfl01525_0010	KleflATP7A	kfl00630_0010	KleflSERCA2	kfl00413_0060
	**Rhodophyta**								
	Bangiophyceae								
		Cyanidioschyzon merolae strai	48a	Cyame1	XP_005539091.1	CyameATP7A	XP_005537873.1	CyameSERCA2	XP_005539258.1
			48b	Cyame2	XP_005535535.1				
		Cyanidioschyzon sp. 5508	49	Cyasp1	C0JV69_9RHOD				
		Galdieria sulphuraria	50a	Galsu1	XP_005706547.1	GalsuATP7A	XP_005703073.1	GalsuSERCA2	XP_005704597.1
			50b	Galsu2	XP_005706047.1				
**Excavata**									
	Percolozoa								
	Heterolobosea								
		Naegleria gruberi NEG-M	51	Naegr1	XP_002673101.1	NaegrATP7A	XP_002678522.1		
	Parabasalia	*Trichomonas vaginalis G3*	52a	Triva1	XP_001307696.1			TrivaSERCA2	XP_001326726.1
			52b	Triva2	XP_001327848.1				
**Amoebozoa**									
		Acanthamoeba castellanii str. I	53	Acaca1	XP_004346011.1	AcacaATP7A	XP_004345382.1		
**SAR**	**Bacillariophyta**								
Stramenopiles	Coscinodiscophyceae								
Alveolata		*Thalassiosira pseudonana CCM*	54	Thaps1	XP_002291807.1	ThapsATP7A	XP_002295670.1	ThapsSERCA2	XP_002286890.1
Rhizaria									
	Bacillariophyceae								
		Phaeodactylum tricornutum C	55	Phathr1	XP_002183586.1	PhatrATP7A	XP_002178233.1	PhatrSERCA2	XP_002183209.1
	**Phaeophyceae**								

		Ectocarpus siliculosus	56	Ectsi1	CBJ30211.1	EctsiATP7A	CBN73914.1	EctsiSERCA2	CBJ48791.1
	**Eustigmatophyceae**								
		Nannochloropsis gaditana CCM	57a	Nanga1	XP_005856121.1	NangaATP7A	EWM23776.1	NangaSERCA2	XP_005853959.1
			57b	Nanga2	EWM25846.1				
	**Haptophyta**								
	Coccolithophores								
		Emiliania huxleyi CCMP1516	58	Emihu1	XP_005767333.1			EmihuSERCA2	XP_005787448.1
	**Ciliophora**								
	Spirotrichea								
		Stylonychia lemnae	59	Style1	CDW79757.1	StyleATP7A	CDW91115.1	StyleSERCA2	CDW82171.1
		Oxytricha trifallax	60	Oxytr1	EJY76435.1|	OxytrATP7A	EJY80608.1	OxytrSERCA2	EJY74000.1
	**Rhizaria**								
		Plasmodiophora brassicae	61	Plabr1	CEO97499.1|	PlabrATP7A	CEO97485.1	PlabrSERCA2	CEO97902.1
**Archaea**	**Euryarchaeota**								
		Candidatus methanoplasma te	62	Mette1	WP_048113480.1	MetteATP7A	WP_048111124.1		
		Methanobacterium paludis	63	Metpa1	WP_013826852.1|	MetpaATP7A	WP_013825732.1		
		Methanocella conradii	64	Metco1	WP_014406891.1	MetcoATP7A	WP_014406977.1		
		Halapricum salinum	65	Halsa1	WP_049994191.1	HalsaATP7A	WP_049992699.1		
		Methanobacterium formicicum	66	Metfo1	AIS32833.1	MetfoATP7A	WP_048084437.1		
		Haloterrigena limicola	67	Halli1	WP_008014917.1	HalliATP7A	WP_008013322.1		
**Bacteria**	**Proteobacteria**								
	alpha								
		Pleomorphomonas koreensis	68	Pleko1	WP_026784876.1	PlekoATP7A	WP_053239844.1		

		Rhodopseudomonas palustris	69	Rhopa1	Q6N3Y0_RHOPA	RhopaATP7A	WP_041803085.1	RhopaSERCA2	WP_013503847.1
		Rhodomicrobium udaipurense	70	Rhoud1	WP_037234292.1				
		Pleomorphomonas oryzae	71	Pleor1	WP_026792736.1	PleorATP7A	WP_036837614.1		
		Methyloceanibacter caenitepia	72a	Metca1	WP_045367820.1	MetcaATP7A	WP_052464462.1	MetcaSERCA2	BAQ18193.1
			72b	Metca2	WP_045367820.1				
		Halocynthiibacter namhaensis	73	Halna1	WP_052245118.1				
		Erythrobacter gangjinensis	74	Eryga1	WP_047007802.1				
		Hyphomonas jannaschiana	75	Hypja1	WP_035580648.1	HypjaATP7A	WP_035578282.1		
		Ruegeria pomeroyi	76	Ruepo1	WP_011048392.1	RuepoATP7A	WP_011046543.1		
		Celeribacter baekdonensis	77	Celba1	WP_009570841.1	CelbaATP7A	WP_040402909.1		
		Parvibaculum lavamentivorans	78	Parla1	WP_012111239.1			ParlaSERCA2	ABS64299.1
		Leisingera caerulea	79	Leica1	WP_027237119.1	LeicaATP7A	WP_027236630.1		
	beta								
		Rubrivivax gelatinosus	80	Rubge1	WP_014428285.1	RubgeATP7A	WP_014429816.1		
		Thauera phenylacetica	81	Thaph1	WP_004380359.1	ThaphATP7A	WP_039920779.1	ThaphSERCA2	WP_004367977.1
		Nitrosospira briensis	82	Nitbr1	WP_025041770.1	NitbrATP7A	WP_025041907.1	NitbrSERCA2	WP_025039952.1
		Paludibacterium yongneupense	83	Palyo1	WP_028536552.1	PalyoATP7A	WP_028536407.1		
		Ralstonia pickettii	84	Ralpi1	WP_024973559.1	RalpiATP7A	WP_004635213.1		
		Nitrosomonas eutropha	85	Niteu1	WP_011633444.1	NiteuATP7A	WP_011634360.1	NiteuSERCA2	WP_011634960.1
		Caldimonas manganoxidans	86	Calma1	WP_019562136.1	CalmaATP7A	WP_019561598.1		
		Azoarcus toluclasticus	87	Azoto1	WP_018990222.1	AzotoATP7A	WP_018991653.1	AzotoSERCA2	WP_026296007.1
		Rhodoferax ferrireducens	88	Rhofe1	WP_011463911.1	RhofeATP7A	WP_011462359.1	RhofeSERCA2	WP_011463963.1
		Burkholderiales bacterium GJ-E	89a	Burba1	WP_045468818.1	RalpiATP7A	WP_051541583.1	BurbaSERCA2	WP_045466804.1
			89b	Burba2	WP_045466347.1	BurbaATP7A	WP_045472466.1		
		Gallionella capsiferriformans	90	Galca1	WP_013293845.1	GalcaATP7A	WP_013292691.1	GalcaSERCA2	WP_013292774.1
	gamma								
		Candidatus Thiomargarita nels	91	Thine1	KHD05613.1			ThineSERCA2	KHD05027.1
		Thioalkalivibrio thiocyanodenit	92	Thith1	WP_018233405.1	ThithATP7A	WP_018233590.1	ThithSERCA2	WP_018233451.1

		Methylomarinum vadi	93	Metva1	WP_031435552.1	MetvaATP7A	WP_031434912.1	MetvaSERCA2	WP_031432430.1
		Methylomicrobium buryatense	94	Metbu1	WP_045226361.1	MetbuATP7A	WP_017839657.1	MetbuSERCA2	WP_017839788.1
		Alcanivorax pacificus	95	Alcpa1	WP_008739734.1	AlcpaATP7A	WP_041025890.1		
		Simiduia agarivorans	96	Simag1	WP_015046314.1	SimagATP7A	AFU97780.2		
		Porticoccus hydrocarbonoclast	97	Porhy1	WP_036858918.1	PorhyATP7A	WP_036861584.1	PorhySERCA2	WP_036859987.1
		Oleispira antarctica	98	Olean1	WP_046008362.1	OleanATP7A	CCK77956.1		
		Zooshikella ganghwensis	99	Zooga1	WP_027709893.1	ZoogaATP7A	WP_051311635.1	ZoogaSERCA2	WP_027709887.1
		Paraglaciecola psychrophila	100	Parps1	WP_007642119.1	ParpsATP7A	WP_007636169.1		
		Hahella ganghwensis	101	Hahga1	WP_026179782.1			HahgaSERCA2	WP_020407242.1
		Saccharophagus degradans	102	Sacde1	WP_011470090.1	SacdeATP7A	WP_011468413.1		
		Azotobacter chroococcum	103	Azoch1	WP_039804127.1	AzochATP7A	WP_039804714.1	AzochSERCA2	WP_039804589.1
		Endozoicomonas numazuensis	104	Endnu1	WP_034832264.1	EndnuATP7A	WP_051786149.1		
		Colwellia psychrerythraea	105a	Colps1	WP_052093663.1	ColpsATP7A	WP_033095258.1		
			105b	Colps2	KGJ93855.1				
		Sedimenticola selenatireducens	106	Sedse1	WP_029132664.1	SedseATP7A	WP_029132122.1	SedseSERCA2	WP_029133537.1
				Sedse2	WP_029134203.1				
		Pseudomonas alcaligenes	107	Pseal1	WP_021703038.1|	PsealATP7A	EQM67165.1	PsealSERCA2	WP_021700187.1
		Marinobacterium jannaschii	108	Marja1	WP_027856026.1	MarjaATP7A	WP_027857548.1	MarjaSERCA2	WP_027858527.1
		Methylococcaceae bacterium 7	109	Metba1	WP_045226361.1	MetbaATP7A	WP_045224079.1		
		Nitrococcus mobilis Nb-231	134	Nitmo1	WP_005004832.1	NitmoATP7A	EAR20729.1	NitmoSERCA2	WP_004999614.1
	delta/epsilon								
		Desulfohalobium retbaense	110	Desre1	WP_015751397.1	DesreATP7A	WP_015752304.1		
		Desulfonatronovibrio magnus	111	Desma1	WP_045214237.1	DesmaATP7A	WP_045212334.1	DesmaSERCA2	WP_045212245.1
		Desulfococcus oleovorans	112	Desol1	WP_012174190.1	DesolATP7A	WP_012175292.1	DesolSERCA2	WP_012176115.1
		Geoalkalibacter ferrihydriticus	113	Geofe1	WP_040100226.1	GeofeATP7A	WP_040097101.1	GeofeSERCA2	WP_040096862.1
		Campylobacter curvus	114	Camcu1	WP_018137173.1				
	zeta								
		Mariprofundus ferrooxydans	115	Marfe1	WP_009850992.1	MarfeATP7A	WP_009849914.1	MarfeSERCA2	WP_009849437.1

	**Actinobacteria**								
		Tetrasphaera australiensis Ben	116	Tetau1	WP_048698213.1				
		Streptomyces fradiae	117a	Strfr1	WP_050363334.1				WP_043470490.1
			117b	Strfr2	WP_045367820.1				
		Streptomyces viridochromogen	118	Strvi1	WP_003987610.1	StrviATP7A	WP_003988287.1		
		Gordonibacter pamelaeae	119	Gorpa1	WP_015540532.1				
	**Firmicutes**								
		Sporolactobacillus vineae	120	Spovi1	WP_010632887.1	SpoviATP7A	WP_010632718.1	SpoviSERCA2	WP_010631526.1
		Desulfitobacterium hafniense	121a	Desha1	WP_011461623.1	DeshaATP7A	CDX04774.1	DeshaSERCA2	WP_026198945.1
			121b	Desha2	WP_005812925.1				
		Pelosinus fermentans JBW45	122	Pelfe1	WP_007954498.1	PelfeATP7A	WP_007960765.1	PelfeSERCA2	WP_007953053.1
		Paenibacillus polymyxa	123	Paepo1	WP_014600014.1	PaepoATP7A	WP_013308458.1	PaepoSERCA2	WP_019687953.1
		Finegoldia magna	124	Finma1	WP_002836101.1	FinmaATP7A	WP_002841204.1		BAG08981.1
		Peptococcaceae bacterium CEE	125	Pepba1	WP_047828246.1	PepbaATP7A	KLU60863.1	PepbaSERCA2	WP_047830305.1
	**Chlamydiae**								
		Criblamydia sequanensis CRIB-	126	Crise1	CDR35322.1	CriseATP7A	CDR35302.1	CriseSERCA2	CDR35275.1
	**Chloroflexi**								
		Sphaerobacter thermophilus D.	127	Sphth1	WP_012872514.1	SphthATP7A	WP_012872295.1	SphthSERCA2	WP_012871833.1
		Ktedonobacter racemifer DSM	128	Ktera1	WP_007911582.1	KteraATP7A	WP_007912812.1		
	**Cyanobacteria**								
		Synechococcus sp. WH 5701	129	SynWH1	WP_037981359.1	SynWHATP7A	WP_006172656.1		
		Cyanobium gracile PCC 6307	130	Cyagr1	AFY29612.1	CyagrATP7A	WP_015108505.1	CyagrSERCA2	WP_015107989.1
	**Nitrospirae**								
		Nitrospira defluvii	131	Nitde1	WP_013250207.1	NitdeATP7A	WP_041186932.1	NitdeSERCA2	WP_013250243.1

	**Gemmatimonadetes**								
		Gemmatimonas aurantiaca (st	132	Gemau1	WP_012682192.1	GemauATP7A	WP_015894714.1		
	**Acidobacteria**								
		Bryobacter aggregatus	133	Bryag1	WP_031497805.1				
