# Supplementary material for: AS3MT-mediated tolerance to arsenic evolved by multiple independent horizontal gene transfers from bacteria to eukaryotes
Source: PLoS One. 2017 Apr 20;12(4):e0175422. doi: 10.1371/journal.pone.0175422 (PMC5398495; doi:10.1371/journal.pone.0175422)
Supplement: S2 Table — In constrained trees, fungi and animal proteins were forced to cluster monophyletically. Statistics of constrained and unconstrained trees were subsequently scored. (PDF) [file pone.0175422.s009.pdf]

S2 Table. Analysis of constrained versus unconstrained trees by RAxML followed by a Kishino-Hasegawa test. In constrained trees, fungi and animal proteins were forced to cluster monophyletically. Statistics of constrained and unconstrained trees were subsequently scored.

**Model, results**	**Statistics**
Fungi	
Model optimization, best Tree: -43318.074364	
Unconstrained Tree: 0<br>Likelihood: -43331.741797 D(LH): -13.667433 SD: 16.463353	Significantly Worse: No (5%), No (2%), No (1%)
Constrained Tree: 1<br>Likelihood: -43724.963619 D(LH): -406.889256 SD: 45.923576	Significantly Worse: Yes (5%), Yes (2%), Yes (1%)
Animalia	
Model optimization, best Tree: -43318.074364	
Unconstrained Tree: 0<br>Likelihood: -43331.741797 D(LH): -13.667433 SD: 16.463353	Significantly Worse: No (5%), No (2%), No (1%)
Constrained Tree: 1<br>Likelihood: -43562.438195 D(LH): -244.363831 SD: 39.839047	Significantly Worse: Yes (5%), Yes (2%), Yes (1%)
