# Supplementary material for: AS3MT-mediated tolerance to arsenic evolved by multiple independent horizontal gene transfers from bacteria to eukaryotes
Source: PLoS One. 2017 Apr 20;12(4):e0175422. doi: 10.1371/journal.pone.0175422 (PMC5398495; doi:10.1371/journal.pone.0175422)
Supplement: S3 Table — (PDF) [file pone.0175422.s010.pdf]

S3 Table
Analysis of AS3MT genomic sequences

**Species**	**Abbreviation**	**Lacks introns**	**Intron-free genes**	**%GC *AS3MT***	**%GC mean**	**%GC stdev**	**%GC diff**	**%GC13 *AS3MT***	**%GC13 mean**	**%GC13 stdev**	**%GC13 diff**	**Introns in genes in close vicinity to AS3MT in the scaffold**
Animalia												
Hydra magnipapillata	*Hydma*	yes	3524 / 24658	32.64	33.01	3.03	no	28.78	32.99	4.55	no	yes
Nematostella vectensis	*Nemve*	No (9 exons)	x	44.29	47.09	5.24	no					
SAR												
Emiliania huxleyi	*Emihu*	No (5 exons)	x	68.74	68.55	4.27	no					
Nannochloropsis gaditana	*Nanga*	No (4 exons)	x	57.07	56.45	6.06	no					
Phaeodactylum tricornutum	*Phatr*	No (2 exons)	x	48.31	51.05	3.77	no					
Thalassiosira pseudonana	*Thaps*	No (3 exons)	x	48.16	47.98	2.79	no					
Fungi												
Trichosporon oleaginosus	*Triol*	yes	1757 / 8602	67.58	63.01	3.87	no	74.23	70.00	5.43	no	yes
Serendipita vermifera	*Serve*	yes	2727 / 15327	55.17	50.81	3.17	no	57.35	53.64	3.76	no	yes
Spizellomyces punctatus	*Spipu*	yes	1509 / 9641	47.65	49.86	2.57	no	51.87	53.13	2.96	no	yes
Schizophyllum commune	*Schco*	No (3 exons)	x	59.53	60.02	3.58	no					
Rhizophagus irregularis	*Rhiir*	No (2 exons)	x	34.9	32.63	5.01	no					
Exophiala aquamarina	*Exoaq*	No (5 exons)	x	48.96	52.11	3.26	no					
Coccidioides immitis	*Cocim*	No (4 exons)	x	51.09	51.33	3.82	no					
Trichophyton tonsurans	*Trito*	No (5 exons)	x	52.77	51.73	4.13	no					
Metarhizium robertsii	*Metro*	No (5 exons)	x	52.07	54.82	5.01	no					
Westerdykella aurantiaca	*Wesau*	no gene in database										
Penicillium expansum	*Penex*	No (5 exons)	x	51.1	52.57	3.49	no					
Aspergillus nidulans	*Aspni*	No (5 exons)	x	51.12	53.47	3.37	no					
Talaromyces cellulolyticus	*Talce*	no gene in database										
Neosartorya fischeri	*Neofi*	No (5 exons)	x	51.5	54.38	3.56	no					
Byssochlamys spectabilis	*Byssp*	No (5 exons)	x	48.12	53.13	3.54	no					
Pseudogymnoascus pannorum	*Psepa*	No (6 exons)	x	60.38	54.01	4.62	no					
