# Supplementary material for: AS3MT-mediated tolerance to arsenic evolved by multiple independent horizontal gene transfers from bacteria to eukaryotes
Source: PLoS One. 2017 Apr 20;12(4):e0175422. doi: 10.1371/journal.pone.0175422 (PMC5398495; doi:10.1371/journal.pone.0175422)
Supplement: S4 Table — (PDF) [file pone.0175422.s011.pdf]

S4 Table
Inventory of AS3MT homologs in organisms that can methylate arsenic

Species/Acc. Nr.	*Protein*	*Identity*	*Coverage*	*E-value*
Homo sapiens				
NP_065733.2	arsenite methyltransferase	100%	100%	0
AAI19639.2	AS3MT protein	100%	97%	0
AAI19638.1	AS3MT protein	99%	97%	0
AAG09731.1	Cyt19 (formally AS3MT)	99%	88%	0
Xenopus tropicalis				
NP_001120227.1	uncharacterized protein LOC100145277	97%	100%	0
XP_002938772.2	PREDICTED: arsenite methyltransferase-like isoform X3	63%	97%	7,00E-166
XP_012823213.1	PREDICTED: arsenite methyltransferase-like isoform X1	64%	95%	2,00E-165
NP_001025616.1	protein-L-isoaspartate(D-aspartate) O-methyltransferase	42%	13%	0.026
XP_012818528.1	PREDICTED: protein-L-isoaspartate(D-aspartate) O-methyltransferase isoform X1	42%	13%	0.037
NP_989324.1	sulfotransferase family, cytosolic, 6B, member 1	29%	13%	9.1
CAJ81528.1	sulfotransferase sult6b1	29%	13%	9.2
Chlamydomonas reinhardtii				
AFS88933.2	arsenite methyltransferase	100%	100%	0
XP_001698772.1	ubiquinone/menaquinone methyltransferase	100%	54%	4,00E-155
XP_001703619.1	ubiquinone/menaquinone methyltransferase	100%	25%	1,00E-60
XP_001701914.1	ubiquinone/menaquinone biosynthesis methyltransferase	34%	28%	9,00E-11
XP_001692723.1	predicted protein	30%	33%	5,00E-06
XP_001698058.1	ubiquinone/menaquinone biosynthesis methyltransferase-like protein	31%	34%	0.001
XP_001694470.1	gamma-tocopherol methyltransferase	29%	28%	0.003
XP_001702747.1	hexaprenyldihydroxybenzoate methyltransferase	47%	12%	0.019
XP_001693971.1	methyltransferase	44%	8%	0.33
XP_001691985.1	hypothetical protein CHLREDRAFT_145375	54%	7%	4
XP_001689913.1	predicted protein	29%	27%	5.6
XP_001691894.1	hypothetical protein CHLREDRAFT_189262	43%	13%	6.2
Cyanidioschyzon merolae				
XP_005539091.1	arsenic methyltransferase Cyt19 [Cyanidioschyzon merolae strain 10D]	100%	100%	0
XP_005535535.1	probable methyltransferase Cyt19 [Cyanidioschyzon merolae strain 10D]	90%	92%	0
XP_005536148.1	hypothetical protein, conserved [Cyanidioschyzon merolae strain 10D]	33%	14%	0.016
XP_005538292.1	hypothetical protein, conserved [Cyanidioschyzon merolae strain 10D]	33%	14%	0.017
XP_005537914.1	probable 24-sterol C-methyltransferase [Cyanidioschyzon merolae strain 10D]	24%	36%	0.033
XP_005539509.1	probable delta(24)-sterol C-methyltransferase [Cyanidioschyzon merolae strain 10D]	26%	29%	0.33
XP_005538298.1	nuclear poly(A) polymerase [Cyanidioschyzon merolae strain 10D]	38%	13%	0.5
XP_005539337.1	unknown methyl transferase [Cyanidioschyzon merolae strain 10D]	39%	10%	2.8
XP_005539084.1	retroelement, alive [Cyanidioschyzon merolae strain 10D]	27%	13%	5.3
Rhodopseudomonas palustris				
WP_011159102.1	arsenite S-adenosylmethyltransferase	100%	100%	0
WP_012496994.1	arsenite S-adenosylmethyltransferase	99%	100%	0
WP_013501552.1	arsenite S-adenosylmethyltransferase	94%	99%	0
WP_047308040.1	arsenite S-adenosylmethyltransferase	94%	99%	0
WP_011503813.1	arsenite S-adenosylmethyltransferase	80%	100%	2,00E-160
WP_011440864.1	arsenite S-adenosylmethyltransferase	84%	94%	6,00E-160
WP_044415711.1	arsenite S-adenosylmethyltransferase	77%	94%	1,00E-143
CAE25999.1	possible methyltransferase [Rhodopseudomonas palustris CGA009]	30%	39%	2,00E-09
WP_042440739.1	methyltransferase	30%	39%	3,00E-09
WP_041801277.1	ubiquinone biosynthesis methyltransferase UbiE	36%	34%	5,00E-09
ABD85953.1	demethylmenaquinone methyltransferase [Rhodopseudomonas palustris BisB18]	36%	34%	6,00E-09
WP_044411109.1	ubiquinone biosynthesis methyltransferase UbiE	28%	53%	7,00E-09
ABJ04429.1	demethylmenaquinone methyltransferase [Rhodopseudomonas palustris BisA53]	36%	34%	2,00E-08
WP_041799373.1	ubiquinone biosynthesis methyltransferase UbiE	36%	34%	2,00E-08
WP_011500643.1	ubiquinone/menaquinone biosynthesis C-methyltransferase UbiE	35%	34%	2,00E-07
WP_011439521.1	ubiquinone/menaquinone biosynthesis C-methyltransferase UbiE	36%	34%	2,00E-07
WP_041802554.1	SAM-dependent methyltransferase	31%	38%	1,00E-06
ABD87006.1	phosphatidyl-N-methylethanolamine N-methyltransferase [Rhodopseudomonas palu	31%	38%	1,00E-06
WP_011662900.1	SAM-dependent methyltransferase	31%	36%	7,00E-06
WP_013499930.1	ubiquinone/menaquinone biosynthesis methyltransferase	33%	34%	1,00E-05

BLASTP (protein-protein BLAST) was run in order to align the AS3MT for each species with other proteins within the same species.
All hits are presented, except for Rhodopseudomonas palustris for which the top 20 hits are presented.
