# Supplementary material for: AS3MT-mediated tolerance to arsenic evolved by multiple independent horizontal gene transfers from bacteria to eukaryotes
Source: PLoS One. 2017 Apr 20;12(4):e0175422. doi: 10.1371/journal.pone.0175422 (PMC5398495; doi:10.1371/journal.pone.0175422)
Supplement: S5 Table — (PDF) [file pone.0175422.s012.pdf]

Supplementary Table S5. *AS3MT* gene and AS3MT protein sequences for selected species with data on arsenic methylation capacity[a].

**Species**	**DNA accession number**[b]	**Protein**[c]
Species that do not methylate arsenic[b]		
Chimpanzee *(Pan troglodytes)*	NC_006477.3 (102093958..102133251)	XP_009457416.1, XP_009457418.1, XP_009457417.1, XP_508007.2
Marmoset *(Callithrix jacchus)*	NC_013907.1 (90264335..90268072)	XP_009008623.1
Species that methylate arsenic		
Human *(Homo sapiens)*	NC_000010.11 (102869453..102901899)	NP_065733.2
Rhesus monkey *(Macaca mulatta)*	NC_007866.1 (102551256..102580628)	XP_001113391.2
Dog (*Canis lupus familiaris)*	NC_006610.3 (15334143..15350749)	XP_005637737.1
Mouse *(Mus musculus)*	NC_000085.6 (46707359..46741099)	NP_065602.2
Chinese hamster *(Cricetulus griseus)*	NW_003614988.1 (266724..294278), NW_006887131.1 (5351..19725)	XP_007649702.1, XP_007637898.1
Rabbit *(Oryctolagus cuniculus)*	NC_013686.1 (50826683..50848325)	XP_008268651.1, XP_008268652.1
Rat *(Rattus norvegicus)*	NC_005100.4 (266482858..266514570)	NP_543166.1
Western clawed frog *(Xenopus silurana tropicalis)*	NW_004668240.1 (23507829..23528700, complement)	NP_001135714.1
Zebrafish *(Danio rerio)*	NC_007112.6 (30974109..30990300)	NP_001034928.1

[a] No data available for guinea pig (*Cavia porcellus*).
[b] Data from NCBI nucleotide database (NCBI nucleotide). The start and end of the gene are presented within brackets.
[c] XP denotes RefSeq records that are derived from the genomic sequence, have varying levels of transcript or protein homology support, and are not subject to further manual curation. We therefore manually curated the amino acids that differed between human AS3MT and other species.
NP denotes RNA and protein products that are mainly derived from GenBank cDNA and EST data and are supported by the RefSeq eukaryotic curation group.
