# Supplementary material for: AS3MT-mediated tolerance to arsenic evolved by multiple independent horizontal gene transfers from bacteria to eukaryotes
Source: PLoS One. 2017 Apr 20;12(4):e0175422. doi: 10.1371/journal.pone.0175422 (PMC5398495; doi:10.1371/journal.pone.0175422)
Supplement: S6 Table — (PDF) [file pone.0175422.s013.pdf]

Supplementary Table S6. Sequence similarities between the human AS3MT protein (NP_065733.2), in whole and methyltransferase domains only, and other species with data on arsenic methylation capacity.

**Species**	**Accession No.**	**Whole protein**			**Methyltrans_31**			**AdoMet_MTases superfamily**		
		Identity[c]	*Coverage*[a]	*E value*[b]	*Identity*	*Coverage*	*E value*	*Identity*	*Coverage*	*E value*
Pan troglodytes	XP_009457418.1	99%	85%	0	99%	100%	1E-108	98%	100%	6E-80
Pan troglodytes	XP_508007.2	99%	100%	0	99%	100%	1E-108	98%	100%	1E-79
Pan troglodytes	XP_009457416.1	98%	100%	0	99%	100%	1E-108	98%	100%	1E-79
Pan troglodytes	XP_009457417.1	98%	85%	0	99%	100%	2E-108	98%	100%	1E-82
Macaca mulatta	XP_001113391.2	97%	100%	0	98%	100%	2E-108	98%	100%	8E-80
Callithrix jacchus	XP_009008623.1	89%	40%	1E-97	89%	52%	4E-48	89%	70%	4E-50
Canis lupus familiaris	XP_005637737.1	86%	84%	0	87%	100%	7E-98	85%	100%	2E-70
Oryctolagus cuniculus	XP_008268652.1	86%	78%	0	85%	100%	3e-98	88%	100%	8e-75
Oryctolagus cuniculus	XP_008268651.1	83%	100%	0	85%	100%	6E-98	88%	100%	8E-76
Cricetulus griseus	XP_007649702.1	78%	96%	0	81%	100%	2E-91	82%	100%	3E-66
Mus musculus	NP_065602.2	77%	100%	0	84%	100%	7E-96	86%	100%	1E-70
Rattus norvegicus	NP_543166.1	76%	100%	0	79%	100%	7E-89	80%	100%	1E-65
Cricetulus griseus	XP_007637898.1	75%	97%	0	81%	100%	7E-92	82%	100%	9E-67
Xenopus silurana tropicalis	NP_001135714.1	61%	97%	5E-166	66%	100%	7E-76	71%	100%	7E-60
Danio rerio	NP_001034928.1	52%	99%	2E-152	64%	100%	2E-75	69%	100%	2E-59

[a] Coverage denotes the percent of the human sequence that overlaps the species sequence.

[b] The Expect value (E) is a parameter that describes the number of hits one can "expect" to see by chance when searching a database of a particular size. Essentially, the E value describes the random background noise. For example, an E value of 1 assigned to a hit can be interpreted as meaning that in a database of the current size one might expect to see 1 match with a similar score simply by chance. The lower the E-value, or the closer it is to zero, the more "significant" the match is.

[c] Identity denotes the percent similarity between the query and subject sequences over the length of the coverage area.
